# Capturing Upper Body Kinematics and Localization with Low-Cost Sensors for Rehabilitation Applications

**DOI:** 10.3390/s22062300

**Published:** 2022-03-16

**Authors:** Anik Sarker, Don-Roberts Emenonye, Aisling Kelliher, Thanassis Rikakis, R. Michael Buehrer, Alan T. Asbeck

**Affiliations:** 1 Department of Mechanical Engineering, Virginia Tech, Blacksburg, VA 24061, USA; aniks@vt.edu; 2 Department of Electrical & Computer Engineering, Virginia Tech, Blacksburg, VA 24061, USA; donroberts@vt.edu (D.-R.E.); rbuehrer@vt.edu (R.M.B.); 3 Department of Computer Science, Virginia Tech, Blacksburg, VA 24061, USA; aislingk@vt.edu; 4 Department of Biomedical Engineering, University of Southern California, Los Angeles, CA 90089, USA; rikakis@usc.edu

**Keywords:** kinematics, inertial sensors, self-supervised learning, sparse sensors, activity recognition, human pose estimation, localization, proximity reporting, Bluetooth beacon, Bluetooth RSS

## Abstract

For upper extremity rehabilitation, quantitative measurements of a person’s capabilities during activities of daily living could provide useful information for therapists, including in telemedicine scenarios. Specifically, measurements of a person’s upper body kinematics could give information about which arm motions or movement features are in need of additional therapy, and their location within the home could give context to these motions. To that end, we present a new algorithm for identifying a person’s location in a region of interest based on a Bluetooth received signal strength (RSS) and present an experimental evaluation of this and a different Bluetooth RSS-based localization algorithm via fingerprinting. We further present algorithms for and experimental results of inferring the complete upper body kinematics based on three standalone inertial measurement unit (IMU) sensors mounted on the wrists and pelvis. Our experimental results for localization find the target location with a mean square error of 1.78 m. Our kinematics reconstruction algorithms gave lower errors with the pelvis sensor mounted on the person’s back and with individual calibrations for each test. With three standalone IMUs, the mean angular error for all of the upper body segment orientations was close to 21 degrees, and the estimated elbow and shoulder angles had mean errors of less than 4 degrees.

## 1. Introduction

### 1.1. Overview

As the US population ages, there is an increasing need for effective and accessible rehabilitation services for debilitating illnesses and injuries such as stroke and degenerative arthritis [1,2]. Effective rehabilitation requires intensive training and the ability to adapt the training program based on patient progress and therapeutic judgment [3]. Telemedicine and telehealth are gaining prominence as avenues for delivering participatory health and wellness in the home at scale. However, a practical approach to physical rehabilitation in the home is not yet possible due to the challenges in capturing meaningful data about how the patient is progressing in a low-cost, easy-to-use way. For upper extremity rehabilitation for stroke survivors, over 30 low-level movement features need to be tracked as the patient performs functional tasks in order to precisely and computationally characterize movement impairment [4]. In addition, detailed activity documentation during daily life is needed to understand the effect of therapy on functional recovery [5]. Although high-end sensing technologies can provide some of the necessary detailed tracking, these technologies are cumbersome even in the clinic and certainly not yet feasible for the home. Tracking of movement through marker-based capture or full-body inertial measurement unit (IMU) systems is impractical and often costly [6,7,8,9,10]. Systems such as exoskeletons or other devices that must be worn along the arm can be cumbersome and may lead to low patient compliance [11,12]. Video or depth camera arrays [13,14] may be objectionable for patients and home occupants due to the feeling of being under constant surveillance [15]. Traditionally, accelerometry has been used to give information about a patient’s motion in a home environment [16,17,18,19], but this provides only coarse measures of patient capability. Some work has also been done in activity recognition in the home [20], or have combined a patient’s location in a home environment with estimates of their activity [21,22,23]; however, quantifying a person’s actual arm kinematics may be more useful than activity recognition.

**Figure 1:**
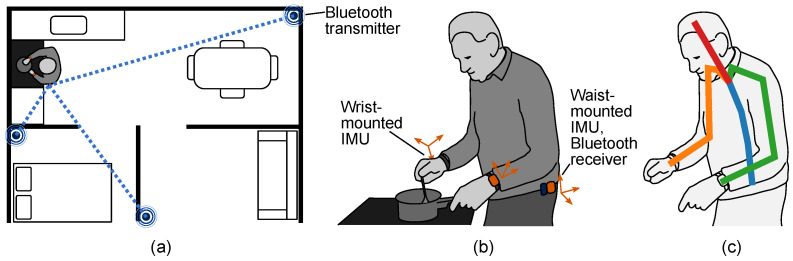
Overview of our strategy for in-home localization and kinematics monitoring. (**a**) In-home localization approach. Bluetooth transmitters are installed around the home, and the received signal strength is monitored at the patient. The localization provides context to the captured activities. (**b**) Minimal sensor set that is unobtrusive during daily life, including IMUs worn on each wrist and the waist, and a Bluetooth receiver worn at the waist. (**c**) Kinematic reconstruction of the torso derived from the worn IMUs.

Consequently, there is a need for low-cost but accurate technologies that can accurately capture a patient’s functional movements during daily life. With kinematics sensing, a patient’s motions can be assessed to monitor progress with rehabilitation. Importantly, contextualizing a person’s motions may be important to determine the circumstances in which they do not use their limbs normally or perform compensatory motions. With this information, therapists could determine the best course of action for rehabilitation. In this paper, we propose a system that can capture both the location of a patient within their home and also their upper body kinematics. As seen in Figure 1, this consists of two components: First, a system based on Bluetooth that can localize the patient within the home (Figure 1a). Second, a system that uses a minimal sensor set to infer the complete upper body kinematics (Figure 1b,c). With these, we present the initial steps towards a practical at-home tele-rehabilitation system.

### 1.2. Background and Related Work on Localization

Location is a crucial context for determining activity. Although the worldwide Global Positioning System (GPS) can provide sub-centimeter level position accuracy, this capability does not extend to indoor scenarios with an absent line-of-sight path from the target to the GPS satellites [24]. Nonetheless, location awareness will still serve as an enabler for indoor health care systems. Location awareness in indoor systems can be enabled by measuring both wireless propagation characteristics (transmitted by known beacons) and motion-related characteristics like acceleration (through accelerometers) and angular velocity (through gyroscopes). The accurate estimation of these characteristics enables location inference.

There are two schools of thought employed in mapping wireless characteristics to location estimates: (1) model-based approaches and (2) fingerprinting-based approaches. In the former, researchers usually assume a wireless propagation model. Subsequently, data points are collected at reference points (RPs) and are used to fit the assumed propagation model. Hence, given observed/estimated wireless characteristics, the distance is readily derived from the assumed propagation model. The difficulty in this approach lies in deriving the appropriate propagation model given that wireless propagation is complex and can vary substantially from location to location [25].

In contrast, fingerprinting-based approaches treat wireless signal measurements as signatures observed in space, frequency, and time. During training, these wireless signatures are observed and intelligently associated with particular locations [25]. After deployment or during testing, new wireless characteristics are observed and compared with previous signatures; and through the association learned during training, the new locations are approximated. The two fundamental building blocks of fingerprinting-based approaches are the association algorithm used and the wireless characteristics selected as signatures.

The most commonly used signature in fingerprinting approaches is the received signal strength (RSS). This commonality is because RSS can be easily obtained from wireless receivers found in phones, Raspberry Pis, and computers [26]. In [26,27,28,29,30,31], an RSS-based fingerprinting database was developed, and the location estimate of a new RSS value was derived as a function of the locations of the *k* most similar RSS values in the database. In [27], the location estimate of the new RSS value was obtained by simply averaging the location of the *k* nearest values in the database. In [26], a Spearman criterion for ranking the *k*-nearest neighbors (*k*-NN) is provided, and the effects of varying *k* on the accuracy of the location estimate are investigated. In [28], the authors recognize that the similarity distance used in prior *k*-NN works incorrectly assumes that similar RSS values translate to similar geometric distances. The authors compensate by proposing a modified feature scaling-based *k*-NN. In [32], a correlation database (CDS) for fingerprinting is built based on the Okumura-Hata propagation model [33]. In that work, to ensure the transmit power is known, the database is built based on transmissions in the control channel. In [34], by relating the locations where RSS signatures are collected to genetic chromosomes, a genetic algorithm [35] is applied to reduce the size of the fingerprinting database.

In addition, artificial neural networks (ANN) have been proposed as fingerprinting-based association algorithms. In [36], a single hidden layer neural network is trained and used to provide location estimates at test time. The neural network has three input nodes, 16 hidden nodes, and two output nodes. The input nodes correspond to the RSS observed at the target from the three access points, and the output nodes provide 2D location estimates. This neural network design provided an accuracy of 1.75 m. In [37], the previous work is extended to a neural network with multiple layers. In that work, the neural network is divided into a data processing section, a denoising section, and a location estimation section. The neural network input is the RSS from the access points, while its output is the 2D location estimate of the target. More recently, a recurrent neural network has been proposed for location estimation [38]. Authors in that work recognize that RSS values received while a target is on a trajectory will be correlated. With this, a recurrent neural network (RNN) enabled trajectory positioning scheme is developed. Recently, a dynamic model estimation technique has been used for indoor positioning [39]. In [40], a chest-mounted IMU is proposed for indoor positioning. In [41], a systematic review is provided for collaborative indoor positioning techniques. A study on indoor positioning systems in harsh wireless propagation environments is presented in [42]. Finally, an automatic context-based positioning system based on Wi-Fi is presented in [43].

Although RSS-aided positioning has been studied rigorously, the requirements for a wireless positioning system in a smart health context are considerably different. For instance, health care professionals are more interested in localizing the subject to a region of interest (RoI) than localizing to the exact coordinates. Hence, for healthcare systems, it will be more suitable to provide proximity reports. Proximity reports reveal how close a subject is close to a set of anchors. A proximity report can be specified by a binary vector y=[1,0,1,0] where the *i*-th element in the vector y specifies whether the subject is in the vicinity of the *i*-th reference node. Clearly, the intersection of the respective vicinity confines the subject to a specific unambiguous RoI. One way to generate proximity reports is by comparing the instantaneous RSS received from different access points to pre-defined RSS thresholds. These thresholds can be derived in a cooperative or non-cooperative fashion. Proximity reports describe the vicinity of the desired subjects without explicitly providing their location estimates. In [44], a campaign is conducted to measure RSS values from different access points at various reference locations. The data collected is used to fit both a linear log-distance model and a Gaussian process regression. Subsequently, the collected data is used to find the optimal threshold for proximity reporting. The selected optimality criterion is the Cramer-Rao bound (CRB). In [45], the work is extended to incorporate multiple thresholds for each reference point. Furthermore, the Barankun bound [46] is used as an optimality criterion. Authors in [47] derive the CRB for a *K*-level RSS quantization scheme. In that work, it is shown that the lower bound on the MSE for proximity location is 50% higher than the bounds in conventional RSS-based systems. Although these prior works are promising, the optimization thresholds are based on propagation models, which may not represent the wireless environments’ actual characteristics. Moreover, most of these works fail to consider the correlation in RSS due to the desired subject trajectory. In order to circumvent the need to assume a model, we propose employing deep neural networks (DNNs) to generate proximity reports. We also propose an RNN to account for the correlation between the current RSS values and previous RSS values. Lastly, as a separate contribution, we validate an already existing algorithm [29] with experimental data. In [29], an improved *k*-NN algorithm was proposed, but was not validated with real-world data. We test the accuracy of that fingerprinting technique proposed for simulated data with real data.

### 1.3. Background and Related Work on Motion Inference

A number of prior works have examined the problem of motion inference via sparse sensors, i.e., predicting the joint angles for the entire body by using only a few sensors.

Several works have used IMUs in conjunction with a video camera [48,49,50,51,52] or depth cameras [53,54]. Generally speaking, the fusion of two different sensor technologies is beneficial; however, for our application, it is impractical and privacy-invasive to use cameras inside a home environment. Another work used RFID tags in conjunction with IMUs to provide more information [55].

A number of recent works have used solely IMUs to reconstruct kinematics [56,57,58,59,60,61]. These have used a variety of approaches for motion inference. Initially, Gaussian processes were used by [56]. Next, ref. [58] used an optimization-based approach, which required knowledge of the person’s initial pose and the sensor locations on their body, with impressive results. More recently, neural networks have been used for motion inference [59,60,61]; these have each used bidirectional long short-term memory (LSTM) neural network architectures [62], where the time history of each sensor provides cues to the current kinematic pose. Both Huang [59] and Yi [61] train their models on the AMASS dataset [63] and the TotalCapture dataset [64] as well as another dataset collected by Huang called DIP-IMU. Their architectures are somewhat similar, but Yi uses a dedicated processing step to estimate ground-foot contacts. Both groups use six IMUs worn on the wrists, lower legs, pelvis, and head, and predict the full-body kinematics.

In our previous work, we used a custom dataset and various numbers of sensors to perform motion inference [60]. The dataset, called the Virginia Tech Natural Motion dataset, contains kinematic recordings of people doing activities of daily living as well as stockers in a warehouse environment. The data was captured with an XSens MVN Link system [65,66] and contains more than 40 h of motion. Using this dataset, we conducted motion inference of both the whole body and the upper body based on 3–6 different body segments. Specifically, we used the XSens-generated orientations and accelerations from each segment to infer the other joints. We note that the orientations and accelerations of the segments are based on the whole-body kinematic reconstruction; thus, their values are somewhat different than if a standalone IMU was placed on each segment. In the present work, we use sparse standalone IMUs to perform kinematics reconstruction. The results are not as good as gold-standard motion capture, but may be sufficient to understand a patient’s motion for rehabilitation.

### 1.4. Contributions

In this paper, we have several contributions. Overall, we present a new strategy for understanding human motion during activities of daily living with just a few unobtrusive sensors, both determining the location of an individual within their home and estimating their kinematics.

In the area of localization, we present new algorithms based on Bluetooth beacons to reduce the uncertainty of a person’s position to an RoI. Our first contribution to both the general area of positioning and in the area of positioning for health care is to develop DNNs for proximity reporting. Similar to existing DNNs for positioning, the neural network tries to learn the nonlinear relationship between the RSS and the target location, but unlike existing DNNs, the neural network does not produce a 2D or 3D location estimate. Instead, the DNN produces a vector that describes the vicinity of the target location. This structure is similar to multilabel classification [67] in machine learning theory, in which a single observed sample can belong to multiple classes. In this paper, we perform simulations to demonstrate the algorithms.

Since healthcare systems use location context to provide recommendations to patients, it is not an absolute necessity to have the exact coordinate of the patients. In this scenario, it is sometimes more important to be able to determine what vicinity the patient is in. Hence, the proposed proximity reporting technique can find application in healthcare systems. However, to have the option of determining the exact coordinates of the patient, our second contribution for positioning estimation is to validate the improved *k*-NN algorithm proposed in the literature [29] with actual Bluetooth beacons. The localization operation in this scenario is divided into training and test stages. During the training stage, the BLE signals from the beacons are collected using a Raspberry Pi [68]. The Raspberry Pi is synchronized with the Beacons and programmed to time stamp the Beacon data and store their RSS values. These RSS values are used to build a fingerprinting database for positioning. During the testing stage, new RSS values are collected and compared with the RSS values in the database. This comparison is used to provide a location estimate. We demonstrate the algorithm with experimental data in a home environment.

In the area of kinematics estimation, we use standalone IMUs combined with our motion inference algorithms [60] to generate an estimate of upper body kinematics during activities of daily living. While several works have examined inferring kinematics of the entire body using sensors on the arms, legs, and torso or head, we use a reduced sensor set with only off-the-shelf sensors on the wrists and pelvis to infer only the upper body. This sensor set is simple, unobtrusive, and easy to use during daily life, especially for people in need of rehabilitation. We compare the accuracy of upper-body kinematic inference using standalone IMUs to information from the ground truth whole-body kinematics. We present the kinematic inference accuracy for each individual joint in the torso since, in rehabilitation contexts, it is useful to understand which joints need additional attention. We also examine the differences in performance between putting the pelvis sensor on the back of the pelvis (as was done previously) versus the side, a location that is more suitable for long-term wear in the home.

The rest of the paper is organized as follows. In Section 2, we present our algorithms and methods for experimental evaluation of localizing a person in a home environment. In Section 3, we present our algorithms and experimental evaluation methods for inferring the kinematics of the upper body. In Section 4, we present all of our experimental results, and in Section 5, we provide the discussion.

## 2. Materials and Methods for Positioning

### 2.1. Overview

In this section, we discuss our localization algorithms and the experimental setup for their evaluation. In the next section, we discuss our kinematics reconstruction algorithms and their experimental evaluation.

### 2.2. Methods for Localization—Proximity Reporting

In this section, we consider a proximity reporting-based technique for indoor positioning, where RSS received from a set of anchors/beacons is compared to predetermined thresholds to determine the position of a target. Note that the actual coordinates of the target is not provided by the proximity reports, the proximity reports only confines the target to a region of interest (RoI). We consider a simulated environment with a set of *U* anchors with known locations in a two-dimensional grid. The locations of the *U* anchors can be defined as:

$$U=\left[\begin{array}{cccc} x_{1} & x_{2} & \cdots & x_{U}\\ y_{1} & y_{2} & \cdots & y_{U} \end{array}\right].$$

The goal is to find the position of a target described with the following vector $s={\left[x,y\right]}^{T}$. Each anchor has a wireless transmitter with Bluetooth 4.0 capabilities that broadcasts ibeacon packets. Each ibeacon packet contains a unique identifier (UUID) that is unique to the broadcasting transmitter. The anchors broadcast at a sampling frequency of 10 Hz, i.e., a single packet is broadcast every 100 ms. A Bluetooth receiver attached to the target collects and stores the packets. The RSS of the signal from each anchor is also stored along with the associated UUID. This UUID differentiates the packets from different anchors. The received power measured in dBm at the target from the *u*th anchor can be characterized as:

(1) $$\begin{array}{cc} r\left(d_{u}\right) & =P_{t}-{\bar{r}}_{}\left(d_{u}\right)+X_{\sigma_{u}}, \end{array}$$

where $P_{t}$ (dBm) is the transmit power of the source, ${\bar{r}}_{}\left(d_{u}\right)$ is propagation loss at a distance $d_{u}$, $d_{u}=∥s-s_{u}∥=\sqrt{{\left(x-x_{u}\right)}^{2}+{\left(y-y_{u}\right)}^{2}}$ and $X_{\sigma_{u}}∼N\left(0,\sigma_{u}\right)$ is a slow-fading term due to shadowing. The propagation loss can be written as:

(2) $$\begin{array}{cc} {\bar{r}}_{}\left(d_{u}\right) & =PL_{u}\left(d_{0}\right)+10\xi_{u}\log\left(\frac{d_{u}}{d_{0}}\right), \end{array}$$

where $PL_{u}\left(d_{0}\right)$ is the path loss measured at a reference distance $d_{0}$, and $\xi_{u}$ is the path loss exponent [25]. Because $PL_{u}\left(d_{0}\right)$ is deterministic, the equivalent mean RSS can be written as:

(3) $$\begin{array}{cc} {\bar{\nu }}_{u} & =PL_{u}\left(d_{0}\right)-{\bar{r}}_{}\left(d_{u}\right),\\ \; & =10\xi_{u}\log\left(\frac{d_{0}}{d_{u}}\right). \end{array}$$

Clearly, ${\bar{\nu }}_{u}$ is dependent on the hub/target position s and the random variable specifying the RSS is given as:

(4) $$\begin{array}{cc} \nu_{u} & ={\bar{\nu }}_{u}+X_{\sigma_{u}}. \end{array}$$

Due to lognormal random variable, the *i*th sample from the *u*th anchor $\nu_{u,i}$ can be characterized by a lognormal distribution:

(5) $$\begin{array}{c} f_{v}\left(\nu_{u,i}\right)=\frac{1}{\sqrt{2\pi }\sigma_{u}}\exp\left(\frac{-{\left(\nu_{u,i}-{\bar{\nu }}_{u,i}\right)}^{2}}{2\sigma_{u}^{2}}\right). \end{array}$$

The model specified by Equations (3)–(5) is used to derive and optimize thresholds in [44,45,69]. However, these thresholds are complex and heavily dependent on the specific environment. To circumvent this challenge, we propose to use a neural network to generate the proximity reports.

#### 2.2.1. Overview of Neural Network

**Figure 2:**
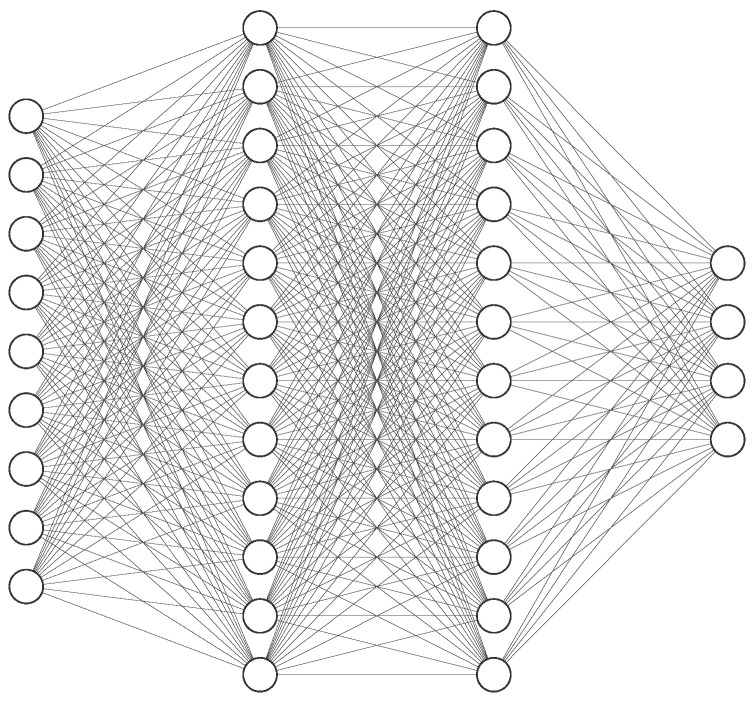
Schematic of a deep neural network with an input layer capable of accepting a nine feature input vector, two hidden layers each with 12 neural network nodes, respectively, and an output layer of 4 nodes.

Deep neural networks act as universal function approximators that can learn the complex relationship between an observation and its label. Given an unknown function, $f^{*}$, that completely describes the observations and their labels in a dataset {S,y}, a DNN tries to learn a set of parameters $\vartheta =\{\vartheta^{1},\vartheta^{2},\cdots ,\vartheta^{Z}\}$ that can produce an approximation of $f^{*}$ as *f*. Here, *Z* represents the number of neural network layers. A simple deep learning network usually has no feedback loop, and its operation can be described as:

(6) $$f\left(s\right)=f^{\left(Z\right)}\left(\cdots f^{\left(2\right)}\left(f^{\left(1\right)}\left(s\right)\right)\right),$$

where $f^{1}$, $f^{2}$, and $f^{Z}$, represent the 1st, 2nd, and *Z*th layers respectively. The operation of the *Z*th layer can be completely described as

(7) $$w^{z}=f_{\vartheta^{z}}\left(w^{z-1}\right)=\Upsilon^{l}\left(W^{z}w^{z-1}+\zeta^{z}\right),$$

where $W^{z}$ and $\zeta^{z}$ describes the weight and bias terms of the *Z*th layer, $w^{z-1}$ describes the output of the previous layer, $\Upsilon^{z}$ denotes the activation function of the *Z*th layer, and $\vartheta^{z}=\left\{W^{z},\zeta^{z}\right\}$ denotes the parameters of the *Z*th layer. Clearly, the operation of the neural network layers can be viewed as a linear transformation empowered by the activation function. In this work, we will restrict our choice of activation functions to the popularized ReLu function. Although DNNs are adept at learning the complex relationships between the input and output, they are not structured to learn temporal correlation. This is intuitive because a plain DNN does not contain any feedback loops, as shown in Figure 2. In order to solve this challenge, recurrent neural networks (RNNs) with built-in loops were developed. These loops allow for information to persist from one time step to another. An RNN is designed to learn the temporal among a sequence of inputs.

**Figure 3:**
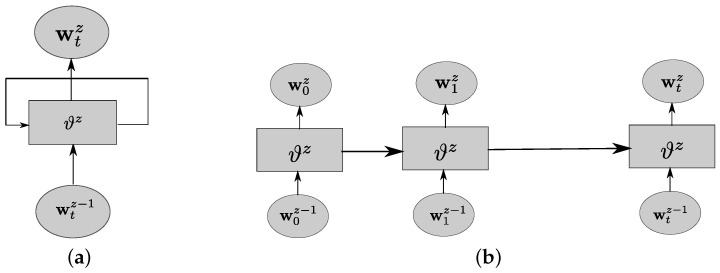
(**a**) A compact representation and (**b**) an unrolled representation of a recurrent neural network.

In Figure 3, the *Z*th layer of the recurrent neural network accepts as input $w_{t}^{z}$, and produces output $w_{t}^{z+1}$. The loop allows for information to be shared across time instances.

#### 2.2.2. System Setup and Data Generation for Proximity Reporting

**Figure 4:**
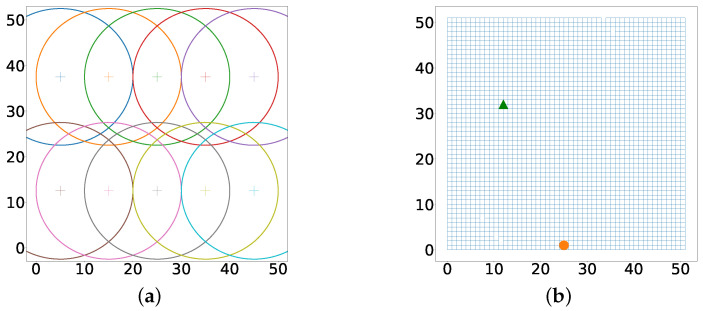
(**a**) Representation of the grid: the vicinity next to an anchor is defined as 15 m from the anchor. (**b**) Simulated patient bounded random walk.

The rest of this sub-section will be focused on developing an indoor positioning system with RSS as the selected wireless propagation characteristic and a recurrent neural network as the selected signature-to-location association function. The output of the neural network is a vector that describes the vicinity of the target. This vector is similar to multi-label classification in image processing [67]. We consider a simulated 50 m by 50 m indoor patient rehabilitation center, which is divided into ten subcenters. An anchor was placed at the middle of each subcenter. Hence, U=10 anchors each equipped with Bluetooth low energy (BLE) beacon transmitters. On entry, the simulated patients were equipped with a mobile hub capable of measuring RSS data from the *U* anchors. The simulated patients were asked to interact with one another and ensure that they are in motion for a particular time interval. The simulated therapists tasked with rehabilitation offer different instructions on physical activities to the simulated patient in the form of push notifications depending on whether they are in the vicinity of certain anchors. Figure 4a shows the grid with 10 access points.

We model the movement of the simulated patient as a bounded random walk from the green arrow to the orange circle. We assume that *U* RSS values sampled from a log-distance model are received at the patient’s hub at every time step. The data received over the time period given by $T_{train}$ is the training data. The log-distance characteristics of each anchor is given in Table A2.

The received RSS values are pre-processed by clipping to ensure that they lie within the range [−100,−50] dBm. The clipping operation can be defined as:

(8) $$\begin{array}{cc} \nu_{u} & =\left\{\begin{array}{cc} -100\;\mathrm{dBm}, & \mathrm{if}\;\nu_{u}<-100\;\mathrm{dBm},\;\\ -50\;\mathrm{dBm}, & \mathrm{if}\;\nu_{u}>-50\;\mathrm{dBm},\;\\ \nu_{u}, & \mathrm{Otherwise}. \end{array}\right. \end{array}$$

#### 2.2.3. Training of Recurrent Neural Network for Proximity Reporting

This section focuses on the training of a long term short memory (LSTM) type of an RNN for proximity reporting. We use a dataset of $T_{train}$ training samples, each with *U* number of features. The features of the *i*th training example can be described as $s_{t,i}=\left\{\nu_{1},\nu_{2},\cdots ,\nu_{U}\right\}$. The dataset is collected offline and each training sample has a label describing the vicinity of the target. Unlike prior works, that use the location estimates as a label, the label is described as a vector y in which its *i*th element is specified as:

$$y^{\left[i\right]}=\left\{\begin{array}{cc} 1, & \mathrm{if}\;\mathrm{the}\;\mathrm{patient}\;\mathrm{is}\;\mathrm{within}\;\mathrm{the}\;\mathrm{vicinity}\;\mathrm{of}\;\mathrm{the}\;i\mathrm{th}\;\mathrm{anchor},\\ 0, & \mathrm{otherwise}. \end{array}\right.$$

**Figure 5:**
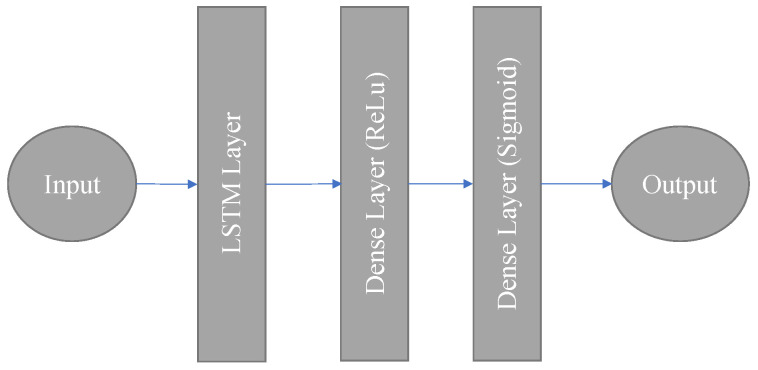
Deployed LSTM neural network for proximity detection.

The input vector $s_{t,i}$ is standardized so all the features lie between 0 and 1. This vector serves as input to the LSTM layers, which has a memory of $T_{LSTM}$ time steps. The neural network is depicted below in Figure 5.

The estimate of the patient’s vicinity, vector $\hat{y}$ can be written as:

$${\hat{y}}^{\left[i\right]}=\left\{\begin{array}{cc} 1, & {\left(w^{Z}\right)}^{\left[i\right]}>0.5,\\ 0, & \mathrm{otherwise}. \end{array}\right.$$

The cross entropy loss function used for training can be written as:

(9) $$L\left(\vartheta \right)=-\frac{1}{T_{train}}\sum_{t=1}^{T_{train}}y_{t}^{T}\log\left(w^{L}\right).$$

With this loss function, and a learning rate, α, the stochastic gradient descent algorithm is used to update the neural network parameters as:

(10) ϑ:=ϑ−α∇L(ϑ).

The training parameters are given in Table 1.

#### 2.2.4. Testing Stage for Proximity Reporting

**Figure 6:**
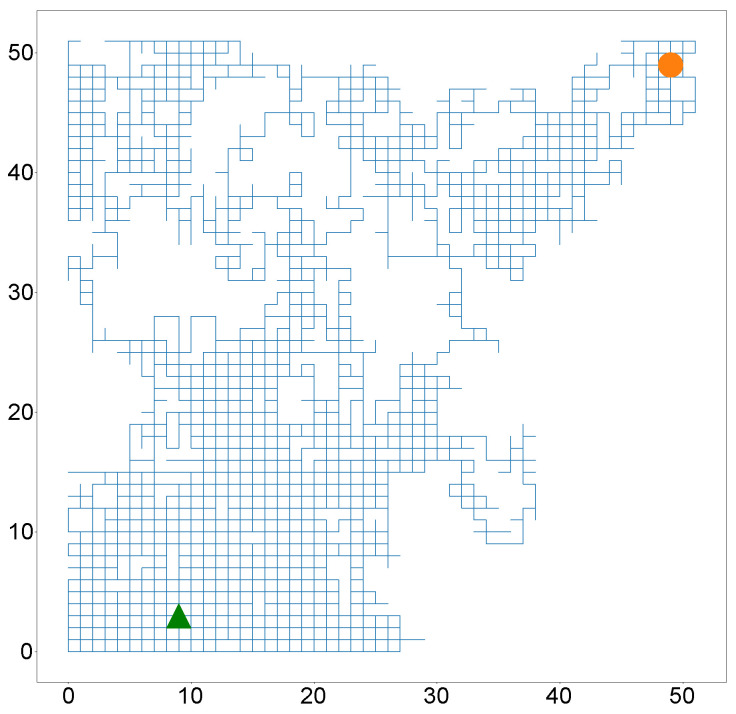
Trajectory used for testing.

To test the proximity detection system, the simulated patient equipped, as previously described, performs another random walk starting from the green arrow and ending at the orange circle as shown in Figure 6.

To show the accuracy of the recurrent neural network, we define the following performance metrics:

- Proximity accuracy: this specifies the indoor system’s ability to detect whether the simulated patient is within a predefined range from the anchor.
- Distance accuracy: this specifies the indoor system’s ability to detect when the simulated patient is not within a predefined range from the anchor.
- Overall accuracy: this specifies the indoor system’s ability to either place the simulated patient within range from the anchor or to determine the absence of the simulated patient within a certain range from the anchor.

### 2.3. Localization with Real Data

In this section, we present a localization technique with real-world data. The RSS is normally distributed in dB and related to the target position. Hence, assigning a unique signature, known as an RSS fingerprint, to different locations is possible. The fingerprint at the target can be described as $G=[\nu_{1},\nu_{2},\cdots ,\nu_{U}]$. The fingerprint is very useful if it varies substantially from one location to another. Fingerprint-based approaches treat RSS as signatures observed in space and time. This fingerprinting operation can be divided into training and testing stages. The training stage involves building a table/codebook with feature vectors and labels. We want to take as little training data as possible, but have it be sufficient to build a good codebook to predict location. The feature vectors are the RSS received from the *U* anchors, while the labels define the target’s (x,y) locations. New RSS values are obtained from the *U* anchors in the testing stage, and a location estimate has to be determined.

#### 2.3.1. Training of RSS Fingerprinting Technique with Real Data

In developing the fingerprinting codebook, we separate an arbitrary area into $R_{m}$ number of rooms. A reference grid is created. Each point in the grid is labeled according to room number and its (i,j)th position in the grid. The (i,j) fingerprint in room *k* is defined as,

(11) $$F_{i,j,k}={\left[\begin{array}{cccc} \nu_{i,j,1} & \nu_{i,j,2} & \cdots & \nu_{i,j,U} \end{array}\right]}^{T}$$

The origin of the coordinate system is the leftmost corner point of the geographical area. The position vector for the (i,j) reference point in room *k* is defined as:

(12) $$D_{i,j,k}={\left[x_{i,j,k}\;\;y_{i,j,k},\;\;z_{i,j,k}\right]}^{T}$$

During the training stage, received signal strength is collected at all the (i,j) points in all *k* rooms. This data collection was carried out for a duration of five minutes at each of the reference points and is averaged in time. The data is stored in a codebook F. The (i,j,k)th entry of the codebook can be accessed as $F_{i,j,k}=F\left[i,j,k\right]$. The signal strength received from the *u*th anchor at the (i,j) reference point in room *k* can be accessed through F[i,j,k][u]. Note that:

$$F_{i,j,k}\left[u\right]=F\left[i,j,k\right]\left[u\right]$$

Similarly, the position vectors are stored in a distance codebook defined as $D=D_{i,j,k}$. The (i,j,k)th entry of the codebook can be accessed as $D_{i,j,k}=D\left[i,j,k\right]$.

#### 2.3.2. Specifications of Area of Interest

We evaluated our *k*-NN-based localization technique in a home environment. The environment had an area of 10.6×7.4 m, which was divided into $R_{m}=7$ rooms. Within this area, four beacons were placed, and 19 reference points were selected. RSS information is collected at each reference point for a duration of five minutes in order to form the signal strength fingerprint. The resulting data at each specific reference point is averaged and placed in a codebook. The figure in Appendix C gives coordinates of the beacon locations and it also provides the positions of the reference points used for building the fingerprinting codebook.

#### 2.3.3. Real-World Validation

For validation, the target was placed at five different test locations and left there for approximately 45 s each. Note that the target is a Raspberry Pi, which is attached to the human body. At each location, RSS is collected at the target from the *U* anchors. The RSS received from each anchor is averaged down to 4 Hz. If no packets are received from the *u*th anchor, the RSS from that anchor is set to $\nu_{u}=\nu_{min}$, where $\nu_{min}$ is the minimum possible RSS. The RSS at the target during testing is defined as:

(13) $$G_{}={\left[\begin{array}{cccc} \nu_{1} & \nu_{2} & \cdots & \nu_{U} \end{array}\right]}^{T}$$

The process of extracting position estimates is described by Algorithms 1 and 2. Algorithm 1 specifies the procedure to determine the closest reference points to the target. These reference points are obtained by comparing the received RSS signatures with the RSS signatures in the codebook. These comparisons are through the Euclidean norm. Note that different RSS signatures in the codebook are associated with different reference points. Furthermore, note that the number of reference points returned (*W*) is a parameter that can be optimized depending on the environment. In this work, W=3 is used. Algorithm 2 returns the centroid of the closest reference points. In this algorithm, the position vector is initialized as a zero vector. Subsequently, the closest reference coordinates are sequentially summed. This cumulative sum is divided by *W*. This centroid is the position estimate of the target.
**Algorithm 1** Generate Closest Fingerprints.  1:∀i,∀j,∀k Compute $∥F_{i,j,k}-G∥$  2:   3:Sort then store the indices of the *W* smallest values in the set W.  4:   5:**return**W

**Algorithm 2** Get Position Estimates Using K-Nearest Neighbors.
**Require:**

$\hat{D}={\left[\begin{array}{ccc} 0 & 0 & 0 \end{array}\right]}^{T}$

  1:   2:
**while** w<W **do**
  3:     4:    Get (i,j,k)=W[w]  5:     6:    $\tilde{D}=D\left[i,j,k\right]$  7:     8:    $\hat{D}=\hat{D}+\tilde{D}$  9:     10:
**end while**
  11:   12:

$\hat{D}=\hat{D}/W$

  13:
**return** $\hat{D}$




## 3. Materials and Methods for Kinematics Estimation

In this section, we discuss our kinematics reconstruction algorithms and the methods for their experimental evaluation. 

### 3.1. Overview

Briefly, we used our dataset and our motion inference algorithms [60] to generate the machine learning models for the upper body motion inference. The full-body kinematics contains information for 23 segments, while the upper body contains information for 15 segments. We aimed to predict the upper body kinematics using only information (orientation and acceleration data) from 3 segments, with the measured upper body kinematics of all 15 segments as the ground truth.

**Figure 7:**
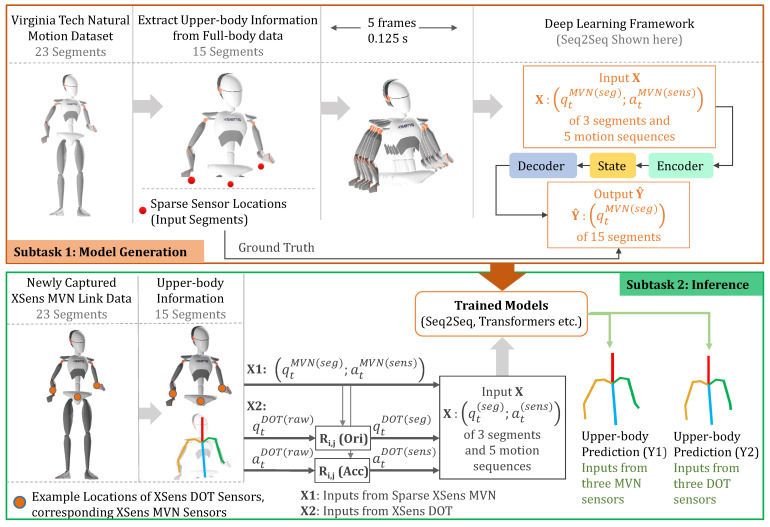
Subtask 1: We used the Virginia Tech Natural Motion Dataset to train our deep learning models. We used three sparse segments (pelvis, right forearm, left forearm), passed five frames of segment orientation ($q_{t}^{MVN(seg)}$) and sensor linear acceleration data ($a_{t}^{MVN(sens)}$) into a neural network, and then predicted upper body segment orientations for those five frames. Subtask 2: For Inference, we used newly captured XSens data (upper body; 15 segments) for the ground truth. We used two sets of sparse inputs: 3 XSens DOT sensors (X1), and 3 XSens MVN segments (X2). The machine learning models from Subtask 1 produced two sets of output upper body kinematics for the two sets of inputs. We then compared the predicted kinematics with the ground truth upper body information from the newly captured XSens MVN Link data. The data used for inference from the MVN was similar to that in Subtask 1; for the DOT sensors, the raw orientation ($q_{t}^{DOT(raw)}$) and acceleration ($a_{t}^{DOT(raw)}$) were calibrated to match the MVN coordinate system by multiplying with a rotation matrix $R_{i,j}$.

A summary of the pipeline for our work is presented in Figure 7. The top of the figure shows how we train our machine learning models. The Virginia Tech Natural Motion Dataset contains kinematic data for the whole body. We extracted just the upper body, and then used motion sequences of orientation and acceleration data from only three segments (pelvis and forearms) as inputs to the machine learning model. The model predicts the orientations of all 15 segments of the upper body, with the ground truth values from the dataset.

Following the creation of the machine learning models, we captured N=4 participants’ full-body kinematics using the XSens MVN Link suit. Simultaneously, we used three XSens DOT sensors (standalone IMUs) to capture orientations and accelerations from the pelvis and forearms. We used the newly captured dataset as the test set for our work: we inferred the predicted upper body kinematics based on (1) orientations from the XSens MVN system for the three sparse body segments (pelvis and forearms), and corresponding sensor accelerations, and (2) orientations and accelerations from the XSens DOT sensors. We compared the inferred upper body kinematics from each of these to the ground truth (15 segments of XSens MVN).

### 3.2. Training Dataset Description

The Virginia Tech Natural Motion Dataset [70] is an enriched dataset of full-body human motion. The data was captured using an XSens MVN Link and includes more than 40 h of unscripted daily life motion in the open world.

The XSens MVN Link suit collects synchronized inertial sensor data from 17 IMU sensors placed in different segments of the body. The data collected from XSens (17 sensors) have reduced magnetic disturbance via a specialized Kalman filter design, and are post-processed to construct accurate human kinematics of 23 segments. The XSens MVN captures full-body kinematics within 5° of absolute mean error compared to an optical motion capture system for various tasks, including carrying, pushing, pulling, and complex manual handling [66,71,72,73,74]. The data includes measurements for segment position, segment linear velocity, both sensor and segment linear acceleration, both sensor and segment orientation, and segment angular velocity and acceleration.

The data were collected from 17 participants, where 13 participants were Virginia Tech students, and 4 were employees of a local home improvement store. Fourteen were male, and three were female. Participants were asked to perform many routine works and material handling tasks, including walking, carrying, pushing, pulling, lifting, and complex manipulation. While generating the deep learning models in this paper, we used orientation and acceleration data of the motion dataset participants {P1, P2, P3, P4, P5, P6, P8, P9, P13, W1, W2, W4} for training, {P10, P12, W3} for cross-validation, and {P7, P11} for testing. Here, ‘P’ refers to Virginia Tech participants, with ranges from P1–P13; ‘W’ refers to workers, with ranges from W1–W4. Details on data collection, data quality, and the role of each participant are documented in [60].

### 3.3. Subtask 1: Model Generation

#### 3.3.1. Training Inputs and Outputs

In this paper, we studied upper-body motion inference, where only the kinematics of the upper body were predicted. We started the training subtask by extracting upper-body information from our training dataset. The XSens MVN Link generates a skeleton of 23 “segments” for the full body, where the first 15 segments are considered as upper body segments. In addition to providing the final body model of 23 segments, the XSens MVN Link also provides the raw data collected from each of the sensors placed on the body. In our previous work [60], we used the orientation and acceleration of sparse segments from the final reconstructed model. Here, we used the linear acceleration of one of the actual sensors from the XSens system (“sensor acceleration”) in combination with the orientation of the reconstructed skeleton segments (“segment orientation”) in our study.

The upper-body inference task was framed as a sequence-to-sequence problem. We entered a sequence of three segment orientations (pelvis, right forearm = RFA, left forearm = LFA) and the corresponding sensor accelerations to predict the orientation of all 15 segments of the upper body over the same sequence. To construct sequences, we downsampled the upper-body orientation and acceleration data from 240 Hz to 40 Hz. We then took five frames of data as both input and output. Five frames of data at 40 Hz corresponds to a motion sequence that is 0.125 s long. Longer input and output sequences add computational complexity to the model without improved results, as discussed in [60]. We apply hyperparameter tuning to maximize neural network performance.

Possible rotational representations of the body segments include Euler angles, rotation matrices, exponential mapping, and quaternions [60]. Euler angle representation has some unavoidable issues namely locking and singularities [75]. Furthermore, rotation matrices incur some computational complexity [59]. An exponential map has been used in many prior works of human motion prediction [76,77,78,79]. However, for representing orientation, we used 4-dimensional quaternions for several well-defined reasons [60].

Before passing the parameters to the model, we normalize the segment orientation and sensor acceleration values of all segments with respect to the root (pelvis) segment. This normalization procedure is the same as in other works, such as [57,59,60]. The root (pelvis) segment orientation with respect to the global frame is $R_{GP}$ (R refers to the orientation, *G* refers to the global reference frame, *P* refers to the pelvis segment reference frame). Then, normalized orientation of any segment with respect to the pelvis segment can be found using the following equation:

(14) $$R_{PB_{i}}={R^{-1}}_{GP}\cdot R_{GB_{i}}$$

In Equation (14), *B* refers to body or segment frame, *i* refers to segment number (ranges from 1 to 15 for the upper body). Thus, $R_{GB_{i}}$ is the *i*th segment orientation with respect to the global frame. The normalized orientation of segment *i* is $R_{PB_{i}}$ (*i*th segment orientation with respect to the pelvis frame). Similarly, the sensor’s normalized acceleration can be found using the following equation:

(15) $${\bar{a}}_{BS_{i}}={R^{-1}}_{GP}\cdot {R_{GB}}_{i}$$

In Equation (15), ${\bar{a}}_{BS_{i}}$ refers to the normalized sensor acceleration for segment *i*. BS refers to the corresponding sensor frame of the segment frame *B*. After normalizing orientation and acceleration using Equations (14) and (15), we zero the mean and divide by the standard deviation of each feature in the training set. Since the validation and test data both simulate unseen data collected in the real-world, we made the assumption that they come from the same underlying distribution as the training data [60].

Briefly, for each task, the input to our model was 5 continuous poses of normalized segment orientation (‘normOrientation’) and normalized sensor acceleration (‘normSensorAcceleration’) for three segments (pelvis, RFA, LFA). The output of the model is the normOrientation value of 15 segments over the sequence of 5 poses.

#### 3.3.2. Deep Learning Models

We used two deep learning architectures for human motion inference: sequence-to-sequence (Seq2Seq) and Transformers [60]. We used the same architectures for inferring upper-body motion from standalone XSens Dot sensors. We chose these architectures because human motion is naturally a temporal sequence, and Seq2Seq and Transformer architectures are efficient for predicting temporal sequences [78,80,81].

Sequence-to-sequence (Seq2Seq) has proven to be successful in neural machine translation [82] and other applications in natural language processing. Seq2Seq models consist of an encoder and a decoder. Furthermore, these models typically contain one or more layers of long short-term memory (LSTM) layers or gated recurrent unit (GRU) layers [62,83]. We also used a variant of the Seq2Seq architecture, where a bidirectional encoder was used [84,85]. Along with the bidirectional encoder we also used Bahdanau attention [85]. This attention mechanism helps to learn the important encoder hidden states.

Similar to the Seq2Seq architecture, a Transformer is also an encoder-decoder-based architecture. It can also be used for human motion inference [86] and other applications in natural language processing [87,88,89,90,91]. Unlike Seq2Seq models, it does not have recurrent layers. We made two models using the Transformer architecture: using a bidirectional encoder, which we refer to as ‘Transformer Encoder’; and using both an encoder and decoder, which we refer to as ‘Transformer Full’. More detail of the Transformer architecture and exact implementation can be found in the original paper [86] and two helpful tutorials [92,93].

In summary, we used two deep learning architectures (1) Seq2Seq (2) Transformers. From these architectures, we prepared four models (algorithms). We refer to these models as (1) Seq2Seq (2) Seq2Seq (BiRNN, Attn.) (3) Transformer Encoder, and (4) Transformer Full.

#### 3.3.3. Training Parameters, Hyperparameter Tuning, and Performance Matrices

We generated the aforementioned models using PyTorch [94]. We conducted hyperparameter tuning using a training and cross-validation set. For each model, we used the same training/validation split. We placed P1, P2, P3, P4 P5, P6, P8, P9, P13, W1, W2, and W4 in the training set (Here, P = Virginia Tech participants and W = worker). In the validation set, we placed P10, P12, and W3. In total, we used 882,452 and 318,484 sequences for training and validation, respectively. We used a V100 GPU and AdamW optimizer with a learning rate of 0.001. Other details of the hyperparameters are provided in Table 2. We used mean absolute error (MAE) as the training loss function.

(16) $$MAE=\frac{1}{mn}\sum_{j=1}^{m}\;\sum_{i=1}^{n}\left|\hat{q_{i}}-q_{i}\right|$$

In Equation (16), $\hat{q_{i}}$ is the predicted segment quaternion, $q_{i}$ is the ground truth segment quaternion, *n* is the number of segments in the body being predicted (15 for the upper body), and *m* is the number of frames in the output sequence (5 frames).

#### 3.3.4. Training Performance Evaluation

For evaluating training performance, we used separate test sets (never used for training or cross validation). Our model evaluation test set came from participants P7 and P11. We used the mean angle difference $\bar{\theta }$ between the ground truth orientation and predicted orientation as a performance matrix of our models. We used the following equation to calculate $\bar{\theta }$ (in degrees).

(17) $$\bar{\theta }=\frac{360}{\pi mn}\sum_{j=1}^{m}\;\sum_{i=1}^{n}\left|<\;\hat{q_{i}},q_{i}\;>\right|$$

In Equation (17), $q_{i}$ is the ground truth quaternion and $\hat{q_{i}}$ is the predicted quaternion for each segment, *i* is the index of the individual body segments, *j* is the index of the frames in the output, *n* is the number of segments (15 for the upper body), and <·,·> is the inner product between two quaternions.

For visualization, we use a forward kinematics solver to plot a line model of the human upper body from the normalized orientation output. The forward kinematics solver uses the segment orientations and then multiplies by a single participant’s segment lengths taken from an XSens MVNX file [60]. We used the following equation to perform forward kinematics given the orientation of the segment:

(18) $${P_{G}}_{i}^{\mathrm{segment}}={P_{G}}_{i}^{\mathrm{origin}}+{R_{\mathrm{GB}}}_{i}\cdot X_{i}^{\mathrm{segment}}$$

In Equation (18), ${P_{G}}_{i}^{\mathrm{segment}}$ is the position of the target segment’s (*i*th segment) endpoint, ${P_{G}}_{i}^{\mathrm{origin}}$ is the position of the origin, and $X_{i}^{\mathrm{segment}}$ is the segment’s length. As before, *G* refers to the global reference frame and *B* refers to the segment’s reference frame.

Although normalization improves generalization, we multiplied by the orientation of the pelvis to view the posture as it would be viewed without normalization for qualitative evaluation. We used the following equation on the all predicted poses:

(19) $$R_{\mathrm{GB}}=R_{\mathrm{GP}}\cdot R_{\mathrm{PB}}$$

### 3.4. Subtask 2: Inference

#### 3.4.1. Test Dataset Overview

As discussed before, we wanted to compare the performance of sparse sensor configurations with three sensors derived from the XSens MVN system versus the performance of standalone sensors. Therefore, we collected data using XSens DOT sensors along with the full XSens MVN Link suit. We collected data from N=4 new participants (2 males, 2 females; ages 23.0 ± 2.3 years). All subjects provided informed consent (Virginia Tech IRB #18-877). After putting on the required sensors, participants were asked to perform some activities of daily living (ADL), listed in Table A1. The data collection was performed in a simulated house environment. Details of the data collection are discussed in the following section.

#### 3.4.2. Data Collection

**Figure 8:**
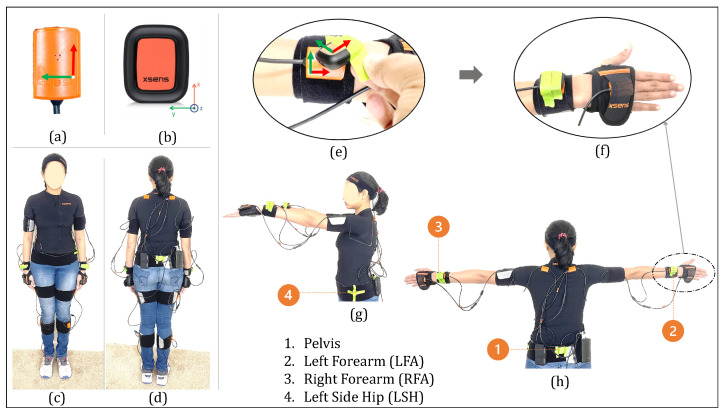
The data collection process using XSens MVN and XSens DOT sensors. In (**a**,**b**), we show the local coordinate system of the XSens MVN sensor and DOT sensors, respectively. In (**c**,**d**), we present front and back views of a participant wearing both sensor systems. In (**e**–**h**) we show detail of the locations of the XSens DOT sensors.

At the beginning of each experiment, the experiment rooms were prepared with the supplies required to perform the activities (full list in Table A1). Then, the participants put on the full XSens MVN Link system. After wearing the suit, four XSens DOT sensors (“DOT sensors”) were secured on top of the Link sensors or the Link suit with tape (see Figure 8). Three DOT sensors were taped on top of the XSens MVN sensors on the pelvis, right forearm, and left forearm; these were sensors that corresponded to the sparse segments in our machine learning framework. The fourth DOT sensor was placed on the left side of the hip, which did not have a corresponding XSens MVN sensor. Complete details of the setup for data collection are presented in Figure 8.

We recorded data with a rate of 240 Hz with XSens MVN and with a rate of 60 Hz with the DOT sensors. The DOT sensors were programmed to collect orientation (quaternions) and acceleration. Later, we downsampled data from both sensors to a rate of 40 Hz and synchronized them manually.

#### 3.4.3. Study Design

**Figure 9:**
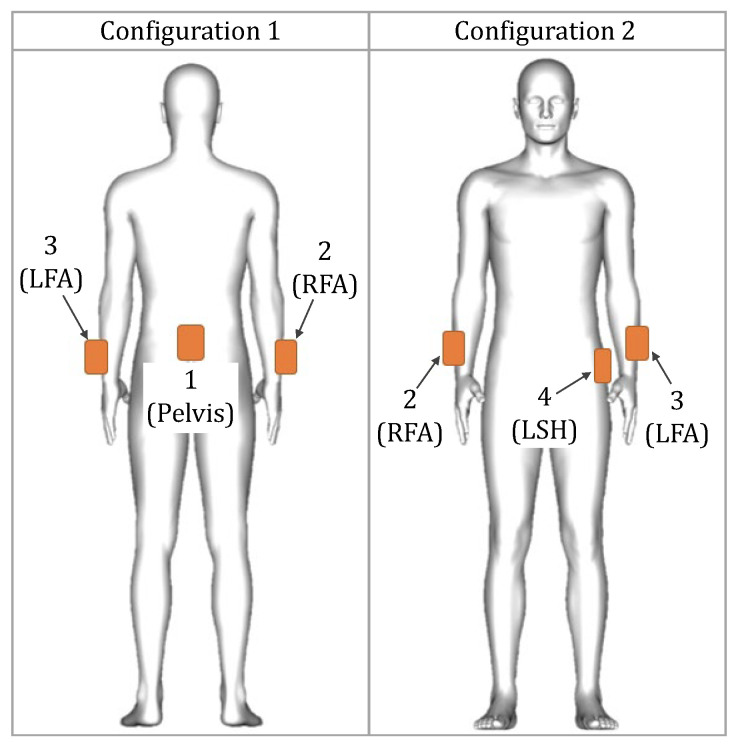
Diagram of the two different possible sensor configurations explored in this paper. Both of them have wrist-mounted sensors in the same locations. On the left, Configuration 1 has the pelvis sensor at the person’s back, which is coincident with the XSens system pelvis sensor. On the right, Configuration 2 has the pelvis sensor at the person’s left hip. LFA = left forearm, RFA = right forearm, SH = left side of hip.

Placing an IMU sensor at the back of the pelvis is quite popular in kinematic inference from sparse sensors (e.g., in [58,59,61]). However, we assumed for practical applications like stroke rehabilitation, that it might be uncomfortable for a patient to wear a sensor on their back for an extended period of time. To investigate solutions to this, we used two configurations to compare the accuracy of upper body inference. As presented in Figure 8, we placed four DOT sensors to formulate two configurations (Figure 9). For Configuration 1, we used DOT sensors at the pelvis, LFA, RFA segments. For Configuration 2, we use a sensor on the left side of the hip (LSH) instead of the pelvis sensor in Configuration 1.

In our study, as the ground truth, we used upper body (15 segments) orientation information from the full XSens MVN suit. We then performed motion inference using (a) three sparse segment configurations derived from the XSens MVN, (b) using three DOT sensors in Configuration 1, and (c) using three DOT sensors in Configuration 2.

#### 3.4.4. Mathematical Framework: Inference Inputs and Outputs, and Sensor Calibration

With the two configurations of the DOT sensors in Figure 9, we mapped the orientation of the three DOT sensors to the three XSens MVN segments (since the segment orientations are inputs to our machine learning models). Similarly, we map the accelerations of the three DOT sensors to the corresponding XSens MVN sensor accelerations. We define two types of mapping functions to translate the DOT measurements to the MVN model, considering two cases: a variable mapping function that is customized for each trial, and a fixed mapping function that is the same across all participants.

For the variable mapping, we mapped the DOT sensor orientation and acceleration in two steps. In the first step, we mapped orientation (DOT sensor to MVN segment), and in the second step, we mapped acceleration (DOT sensor to MVN sensor). For orientation mapping, we assumed that a fixed rotation matrix (mapping function) existed between the DOT sensors and corresponding XSens MVN segment for each individual recording session. Similarly, for acceleration mapping, we assumed that a fixed rotation matrix existed between each DOT sensor and the corresponding XSens MVN sensor.

That means the mapping functions (orientation and acceleration) on a particular day (or recording session) may not be the same as the next day. We made this assumption because, for each recording session, the XSens MVN system performs a local calibration. This calibration might be different for a different recording session. We mapped the orientation and acceleration of DOT sensors to XSens MVN using the following equations:

(20) $$R_{i,j}\left(\mathrm{Ori}\right)={\left({R_{i,j}}^{MVN(seg)}\right)}_{n}\cdot {{\left({R_{i,j}}^{DOT(raw)}\right)}_{n}}^{-1}$$

(21) $$\left({R_{i,j}}^{DOT(seg)}\right)=R_{i,j}\left(\mathrm{Ori}\right)\cdot \left({R_{i,j}}^{DOT(raw)}\right)$$

(22) $$R_{i,j}\left(\mathrm{Acc}\right)={\left({R_{i,j}}^{MVN(sens)}\right)}_{n}\cdot {{\left({R_{i,j}}^{DOT(raw)}\right)}_{n}}^{-1}$$

(23) $$\left({a_{i,j}}^{DOT(sens)}\right)=R_{i,j}\left(\mathrm{Acc}\right)\cdot \left({a_{i,j}}^{DOT(raw)}\right)$$

In Equations (20)–(23), $\left({R_{i,j}}^{MVN(seg)}\right)$ and $\left({R_{i,j}}^{MVN(sens)}\right)$ are the MVN segment orientation and MVN sensor orientation of the *i*th segments (pelvis, LSH, LFA, RFA, etc.) from the *j*th recording session. These values are rotation matrices corresponding to the orientations (quaternions) of each segment. Similarly, $\left({R_{i,j}}^{DOT}\right)$ is the orientation of a DOT sensor and $\left({a_{i,j}}^{DOT}\right)$ is the linear acceleration from a DOT sensor. Values with (raw) superscripts are the raw DOT sensor data, while values with (seg) and (sens) have been calibrated to match the MVN segment and sensor data, respectively. For both MVN and DOT data, values with *n* subscripts were those corresponding to a particular frame *n* that we used for calibration. $R_{i,j}\left(\mathrm{Ori}\right)$ and $R_{i,j}\left(\mathrm{Acc}\right)$ are the desired orientation and acceleration calibration mapping functions (rotation matrices), respectively, for the *i*th segment and *j*th recording session.

Synchronization of the DOT sensor and XSens MVN is crucial to determine the mapping functions $R_{i,j}\left(\mathrm{Ori}\right)$ and $R_{i,j}\left(\mathrm{Acc}\right)$. For synchronization, we first downsampled the DOT sensors and corresponding XSens MVN segments to a frequency of 40 Hz. We then carefully synchronized both sensor data with a standard starting and ending frame based on a sudden bump, which is visible in the acceleration data. To then find the orientation mapping function $R_{i,j}\left(\mathrm{Ori}\right)$, we picked a random single frame (n), took the value of ${\left({R_{i,j}}^{MVN(seg)}\right)}_{n}$, and multiplied (matrix product) it with the inverse of the corresponding DOT sensor orientation ${{\left({R_{i,j}}^{DOT(raw)}\right)}_{n}}^{-1}$. This is shown in Equation (20). Similarly, to find the acceleration mapping function $R_{i,j}\left(\mathrm{Acc}\right)$, we used the same frame *n*, took the value of ${\left({R_{i,j}}^{MVN(sens)}\right)}_{n}$, and multiplied (matrix product) it by ${{\left({R_{i,j}}^{DOT(raw)}\right)}_{n}}^{-1}$. This is in Equation (22).

Once we constructed the fixed mapping functions, we then used these mapping functions for all of the data collected in that session (Equations (21) and (23)), to map the orientation and acceleration of all frames of the XSens DOT sensor to XSens MVN coordinate system. Finally, we used the mapped data (${R_{i,j}}^{DOT(seg)},{a_{i,j}}^{DOT(sens)})$ as the input to the models. These relationships can also be seen in Figure 7.

For the fixed mapping, we assumed that a fixed rotation matrix (mapping function) exists between the DOT sensors and the corresponding XSens MVN segment and sensors, irrespective of the recording session. In other words, we assumed there exists a constant universal mapping function between DOT sensors and XSens MVN (sensors and segments). We made this assumption to investigate a generalized approach to using standalone IMUs for human motion inference. We found this fixed mapping function by averaging the variable mapping functions, using Equation (24).

(24) $$\bar{R_{i,j}}=quaternion\_average\left(R_{i,j_{1}},R_{i,j_{2}},R_{i,j_{3}},\ldots \right)$$

**Figure 10:**
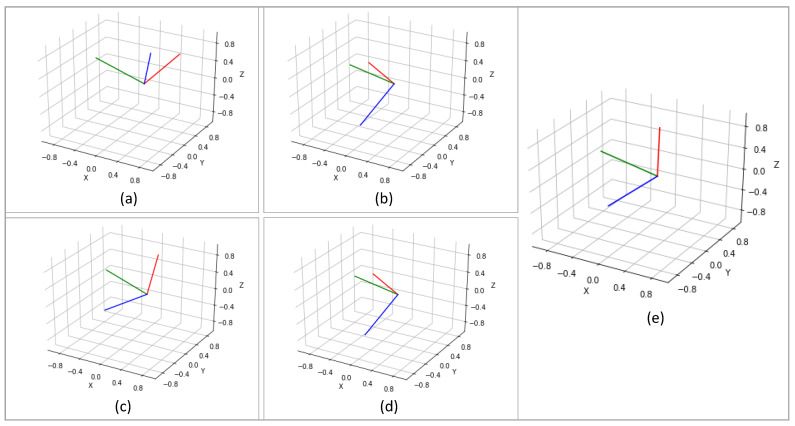
In (**a**–**d**), we plot the rotation matrix (variable mapping function) of XSens DOT sensors to MVN sensors for different persons on different recording sessions (only right forearm sensor is shown here). In (**e**), we plot the fixed rotation function (fixed mapping function). The fixed mapping function (using Equation (24)) is the quaternion average of the other four rotations. In each graph, the red, green, and blue lines correspond to the *x*, *y*, and *z* axes of each rotation matrix.

In Equation (24), we simply average the mapping functions of different recording sessions using the quaternion averaging method [95]. We then used $\bar{R_{i,j}}$ (refers to both the orientation and acceleration mapping functions) to map all DOT sensor data to XSens MVN data. In our study, we averaged the variable mapping functions from j=4 recording sessions to estimate the fixed mapping function. Figure 10 shows the individual rotation matrices (${R_{i,j}}^{MVN(sens)}$) for each of the j=4 recording sessions and the average rotation matrix from these four quaternions.

Inference using the sparse configuration of XSens MVN was straightforward. We used the segment orientation and sensor acceleration information of three sparse segments from the newly collected data to predict the upper body using the four machine learning models. However, for inference with standalone DOT sensors, we considered all possible combinations of the factors: deep learning models could be {Seq2Seq, Seq2Seq (BiRNN, Attn), Transformer Enc., Transformer Full}; the DOT sensors could be in {Configuration 1, Configuration 2}; and the Mapping Function could be {Variable Mapping, Fixed Mapping}.

## 4. Results

### 4.1. Localization Results: Proximity Reporting in Simulation

**Figure 11:**
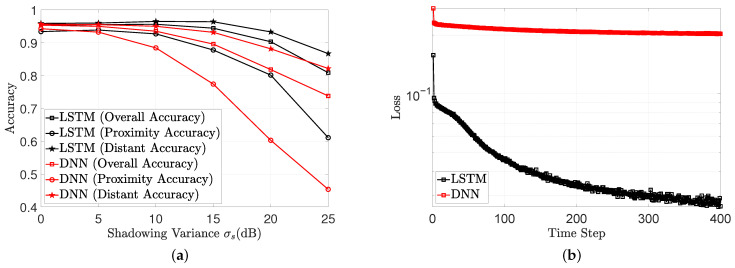
(**a**) Accuracy versus shadowing variance. (**b**) Training loss versus time step.

As simulation validation, we show the ability of the trained recurrent neural network (a DNN with a single LSTM layer) to withstand highly variable data; we also present the performance of a plain DNN. The anchor characteristics are presented in Appendix B. The path loss $PL_{u}\left(d_{0}\right)$ and path loss exponent $\xi_{u}$ in that table describes the characteristics unique to a specific anchor. In Figure 11a, as the shadowing variance increased, $\sigma_{s}$, the accuracy of the LSTM degraded more slowly than with a simple DNN. More specifically, at a variance of $\sigma_{s}=15$ dB, the proximity accuracy of the DNN was 78%, while the proximity accuracy of the LSTM was 87%. At the same variance, the distance accuracy of the DNN and LSTM was 89% and 95%, respectively. The training loss presented in Figure 11b indicates that the LSTM might have better performance since it converges to a smaller loss value.

### 4.2. Localization Results: Proximity Reporting with Real World Data

**Figure 12:**
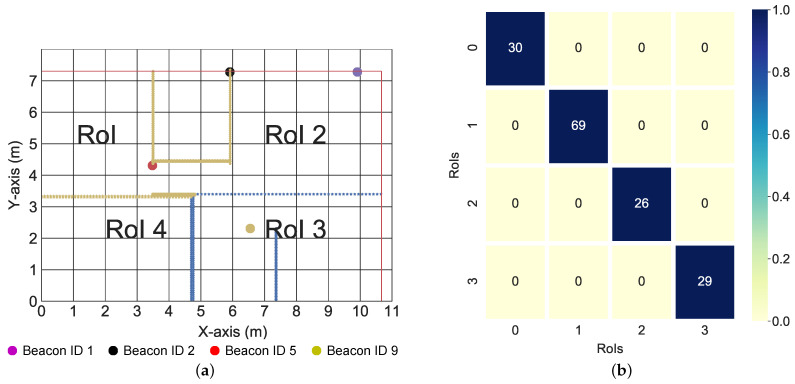
(**a**) Floor plan, showing the beacon locations and the regions of interest; (**b**) confusion matrix showing accuracy of neural network-based proximity reporting.

In this section, we validate the model-free neural network proximity reporting system with real data. The considered environment is an area of 10.6×7.4 m. The reference points and the beacons are placed as described in Section 2.3.2. The beacons and reference coordinates are also shown in Appendix C. However, unlike in Section 2.3.2, the area was divided into four regions of interest, as shown in Figure 12a. The red line indicates the wall separating the indoors and the outdoors. The yellow lines indicate walls separating various indoor regions, while the blue lines indicate a separation from one indoor region to the next without a wall. A three layered LSTM was trained to recognize each RoI. There were four inputs to the LSTM, each representing the mean RSSI values measured over 0.5 s from each of the four beacons. There were also four LSTM outputs, each representing the four RoIs. From Figure 12b, the LSTM was able to perfectly determine when the target was in each RoI.

### 4.3. Localization Results: Positioning with Real World Data

**Figure 13:**
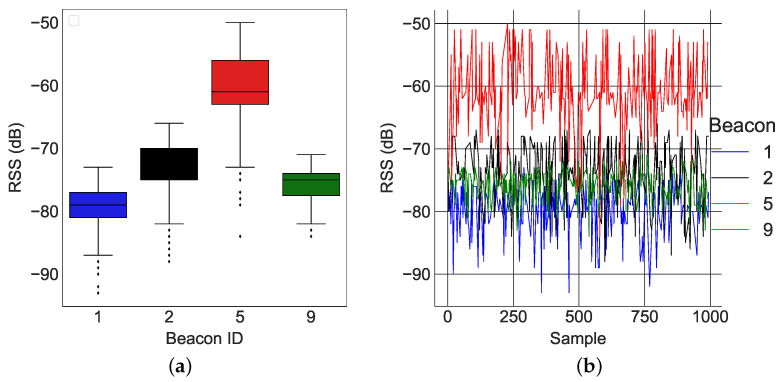
RSS at Reference point 1. (**a**) Distributions of RSS for different beacons; (**b**) RSS versus sample number (samples are taken at 4 Hz).

**Figure 14:**
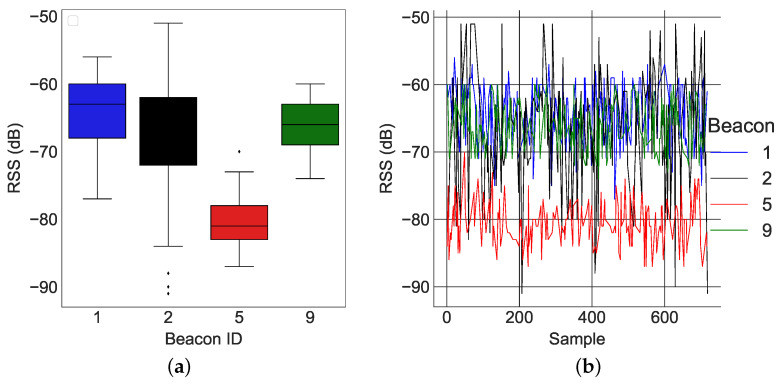
RSS at Reference point 11. (**a**) Distributions of RSS for different beacons; (**b**) RSS versus sample number (samples are taken at 4 Hz).

In this section, we present real-world results using the *k*-NN algorithm presented in [29]. Two examples of RSS data at different locations are shown in Figure 13 and Figure 14. The average values of the RSS were used to form the codebook for localization.

**Figure 15:**
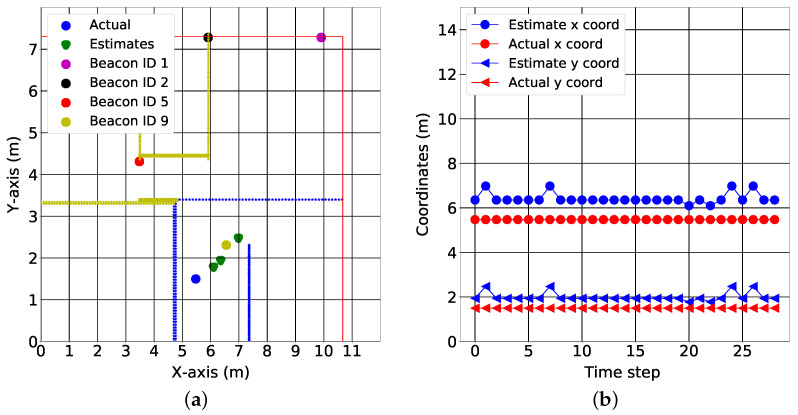
Testing in the kitchen. (**a**) Floor plan view, and (**b**) location estimates over time.

**Figure 16:**
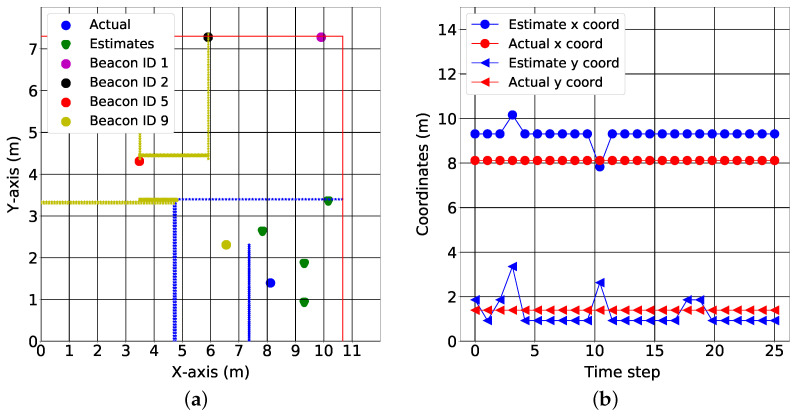
Testing in the dining room. (**a**) Floor plan view, and (**b**) location estimates over time.

**Figure 17:**
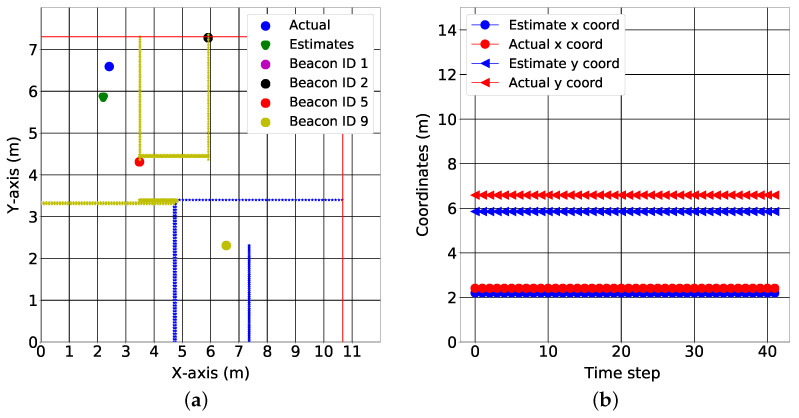
Testing in a bedroom. (**a**) Floor plan view, and (**b**) location estimates over time.

We provide test results for data collected in three of the seven rooms, as shown in Figure 15, Figure 16 and Figure 17. The colored lines in these figures represent the demarcations separating the RoIs. These demarcations also affect the RSS in the form of shadowing. While the minimum mean square error of the position estimates was 1.78 m, the figures show that the target can be localized to an RoI. More specifically, the fingerprinting technique with real world data can predict what region of the house the target is located. This is crucial for smart health applications where knowing the section of the house that different motions are performed in gives cues about the purpose of those motions and which activities of daily living might need additional rehabilitation.

Figure 15b, Figure 16b and Figure 17b show the variation of the estimates over time in both the x coordinates and y coordinates. It is important to note the relationship between the spatial and the temporal view. For the kitchen, the algorithm produced varying estimates while the target was positioned at a fixed coordinate (see Figure 15a); this variation was captured best in the temporal view. In the temporal view (see Figure 15b), the estimates varied over the time step. This trend was also observed in the dining room (see Figure 16a,b). However, in the final test location (bedroom 2), the algorithm produced a stable estimate (see Figure 17a). This stability was validated by the temporal view in Figure 17b.

### 4.4. Motion Inference Results

Here, we first provide results on the performance of our algorithms. We describe the quantitative results for our algorithms, then we show the visualization of a few postures predicted by the models.

#### 4.4.1. Quantitative Analysis

With the quantitative results, we first present the inference performance of our models using the VT Natural Motion Dataset. We then describe how the trained models performed with the new dataset using sparse segments of XSens MVN. Finally, we present the performance of our models using DOT sensors, considering the two configurations and the two mapping functions.

##### Test Performance Evaluation Using Sparse Segments of XSens MVN

**Figure 18:**
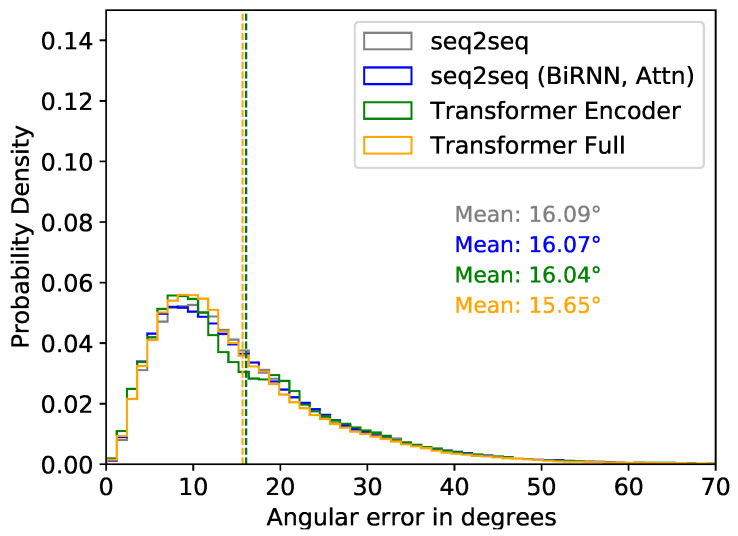
Distribution of the mean angular error for motion inference using the sparse segments of XSens MVN (from the new dataset).

We first present results using our new test set, as described in Section 3.4.1. Here, we expect similar results to our prior work [60], since we used the sparse data from XSens MVN. In Figure 18, we plot the angular error distribution combining all predicted segments for all four models, including the mean angular error for all models.

##### Test Performance Evaluation Using XSens DOT Sensors

**Figure 19:**
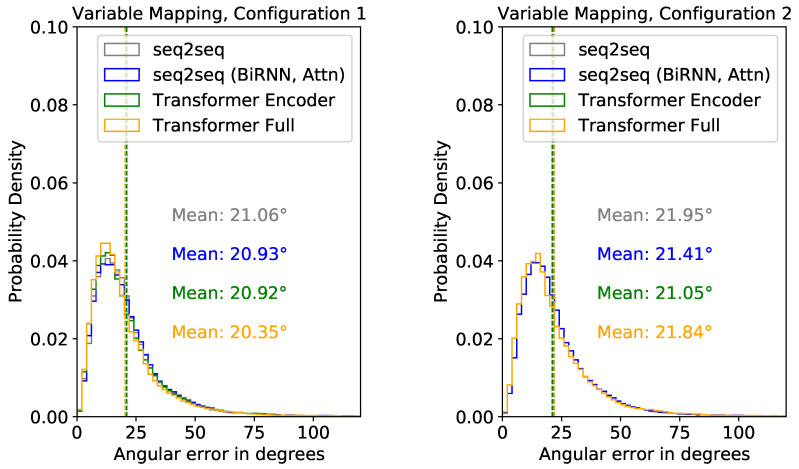
Angular error distribution of motion inference using Xsens DOT sensor with varying configurations (from the new dataset). Configuration 1 had the pelvis sensor on the back of the pelvis, next to the XSens sensor; Configuration 2 had the pelvis sensor on the side of the pelvis.

Next, we present inference results using the XSens DOT sensors considering different factors. Figure 19 shows the distribution of the mean angular error of the predicted segments relative to the ground truth segments for the two configurations we described in Figure 9. We consider the variable mapping function for the results shown in Figure 19. Overall, all the models performed similarly in both Configurations 1 and 2. However, results were slightly better in Configuration 1. Therefore, comparing the results of all configurations with the variable mapping, we used Configuration 1 and the Transformer Full model for further analysis, as these had the best results.

In Table 3, we present the results from the DOT sensors using the fixed mapping function. Here, average results were much better in Configuration 1 than Configuration 2. In Configurations 1 and 2, the transformer models had the minimum mean angular error, with values of ∼33° in Configuration 1 and ∼43° in Configuration 2.

##### Comparison of Segment-Wise Mean Angular Error of Predictions by XSens MVN and XSens DOT Sensors

**Figure 20:**
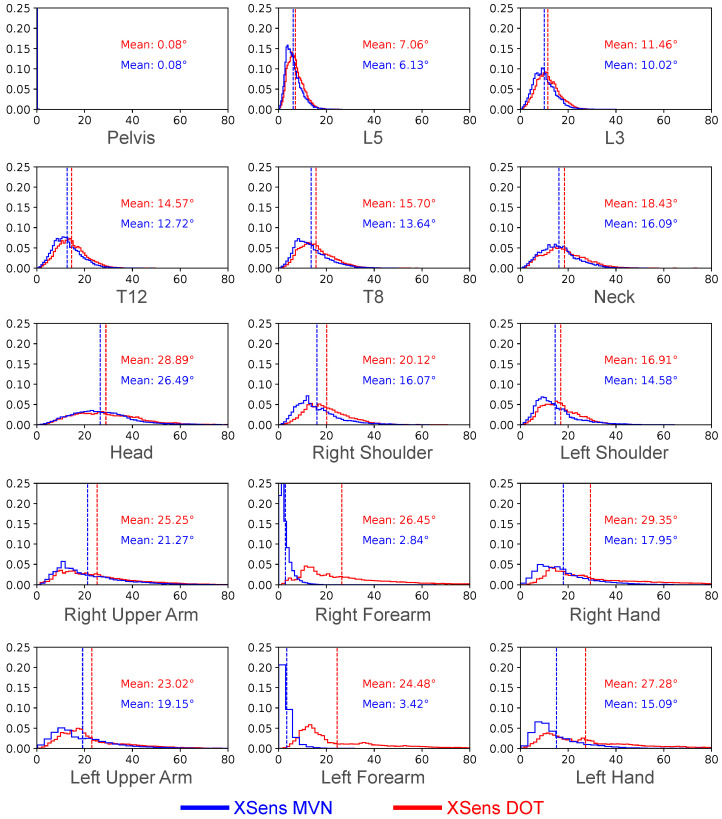
Segment orientation errors for each segment in the XSens model. *X*-axis is angle in degrees. Blue histograms show the angular error distribution for the MVN segments, while red histograms show the angular error distribution using the DOT sensors. The mean error is shown in each case.

In Figure 20, we compared inference results of the DOT sensors with results from sparse XSens MVN segments. We only compared the performance of the Transformer Full model. For DOT sensors, we present results with the variable mapping function and Configuration 1. There are 15 sub-figures for the 15 upper-body segments. For both XSens MVN and XSens DOT, we plot the mean angular error distribution for each segment relative to the ground truth. While the overall minimum mean angular error of prediction using XSens MVN and XSens DOT are ∼15.65° and ∼20.35°, respectively, for the Transformer Full model (see Figure 18 and Figure 19), Figure 20 shows how these errors are distributed among the segments.

In most cases, the two inputs gave similar results, but using sparse segments of the XSens MVN performed several degrees better. Both inputs had relatively low mean angular errors for the first six segments (Pelvis, L5, L3, T12, T8, Neck), and the MVN inputs had low errors for the right and left forearms. For the XSens MVN, the maximum mean error occurred for the ‘Head’ segment, ∼29°. Noticeably, the XSens DOT had much higher mean errors for inferring motions of the ‘Left Forearm’ and ‘Right Forearm’, with errors of ∼24° and ∼26°, respectively; for comparison, the MVN inputs had errors of ∼3°.

**Figure 21:**
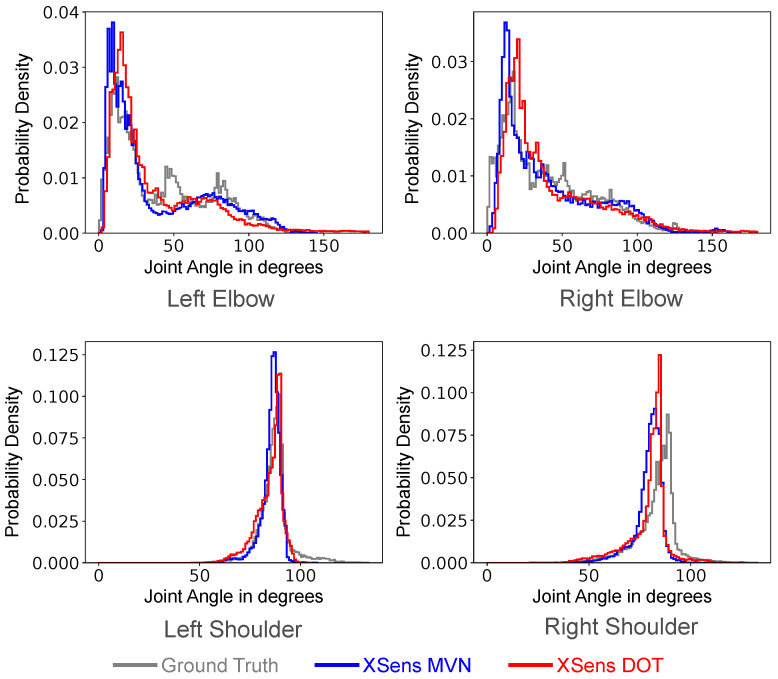
Joint angle distribution for selected joints. The blue histogram shows the joint angle distribution for the MVN segments, while the red histogram shows the joint angle distribution using the DOT sensors (variable mapping, Configuration 1). The grey histogram presents the ground truth for the corresponding joint angles. For the elbow, 0° corresponds to the arm straight.

We next computed histograms of the distribution of the joint angles measured in the test set (Figure 21). Specifically, we plot the left and right elbows and left and right shoulders. To find the joint angles, we took the angle between the two quaternions for the segments on either side of the joint. Therefore, the elbow angles were computed via the angle between the upper arm and forearm, while the shoulder angles were computed by the angle between the T8 segment (near the upper chest) and the upper arm. Note that this method finds the smallest angle between the two quaternion orientations, so we do not distinguish between the three different degrees of freedom at the shoulder. Notably, both the XSens MVN and DOT inputs gave joint angle distributions very close to the ground truth for all of the angles investigated.

**Figure 22:**
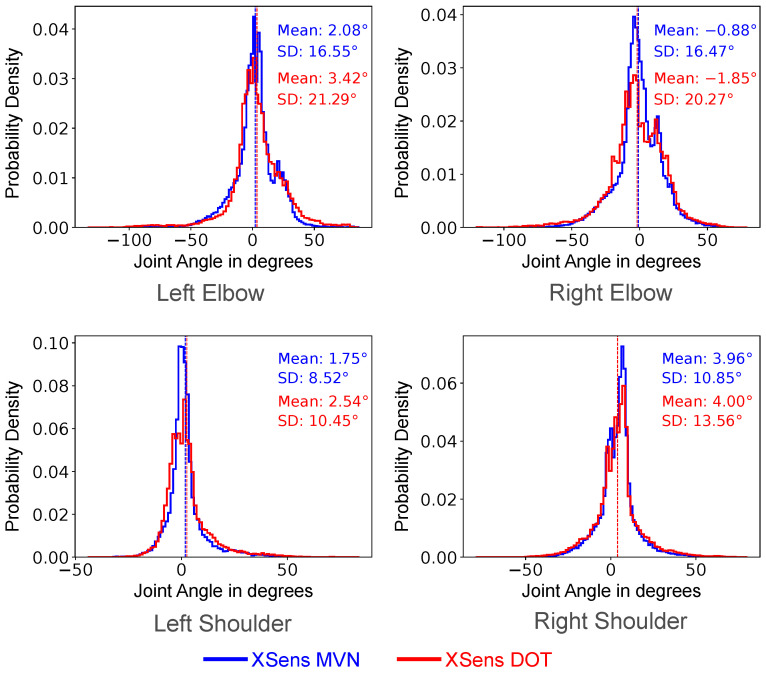
Joint angle error distribution for selected joints. The blue histogram shows the error distribution for the MVN segments, while the red histogram shows the error distribution using the DOT sensors (variable mapping, Configuration 1). The mean error and standard deviation are shown for each distribution.

Next, we computed histograms of the error between the ground truth joint angle and the joint angles predicted by either the MVN sparse segments or the DOT sensors (Figure 22). To find these values, we took the ground truth joint angles (as computed above) and subtracted from them the inferred joint angles (from the MVN and DOT sensors separately). In these graphs, negative values indicate that the inferred angle predicted a more acute angle than the ground truth. For the shoulder, negative values indicated that the arm was closer to the side than the ground truth. In each case, the mean joint angle error was less than 4.0° for both the MVN sparse sensors and the DOT sensors. The error distributions were approximately symmetric around zero in both cases.

#### 4.4.2. Qualitative Analysis

Qualitative evaluation is performed in most of the studies of human motion inference [48,59,96] to give intuition into how well the reconstruction performs. Quantitative measures help analyze different aspects of the models’ performance, and a visual evaluation is necessary to build intuition for how the models make predictions. We only evaluated a few poses to demonstrate our work.

**Figure 23:**
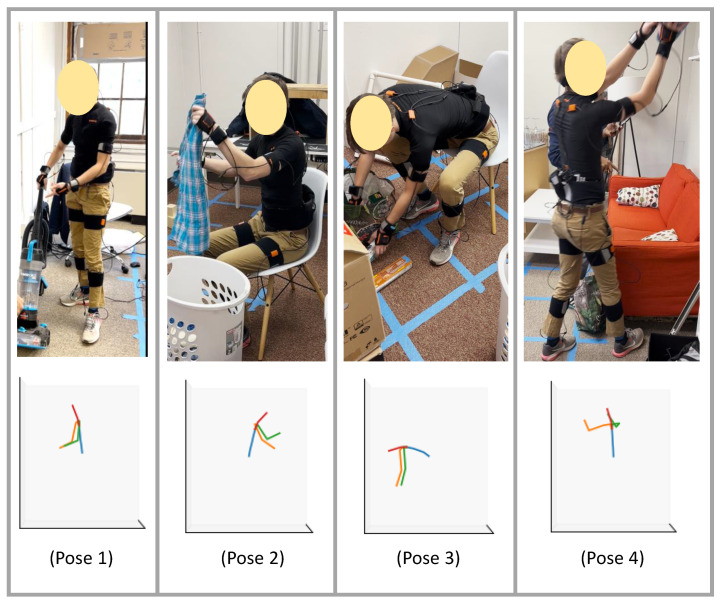
Figures in the first row are the samples of actual human poses during the different ADL tasks listed in Table A1. These include: vacuum cleaning (pose 1), folding laundry (pose 2), picking grocery items (pose 3), and putting objects on a high shelf (pose 4). In the second row, we present the skeleton model of the ground truth for the upper body for similar poses. The ground truth is reconstructed using the XSens MVN Link system. The actual human poses in the first row look slightly different than the ground truth poses in the second row because the photos correspond to slightly different times than the ground truth poses.

Figure 23 presents four sample poses and the ground truth reconstructed using the XSens MVN Link system. We note that the actual human poses in Figure 23 correspond to slightly different times than the stick figures. The poses are representative of the activities listed in Table A1. The first pose shows vacuum cleaning, and the second pose shows folding laundry. The third pose is from organizing groceries, which is similar to picking something up from the ground. The fourth pose illustrates placing an object (either grocery/laundry) on a higher-level shelf.

**Figure 24:**
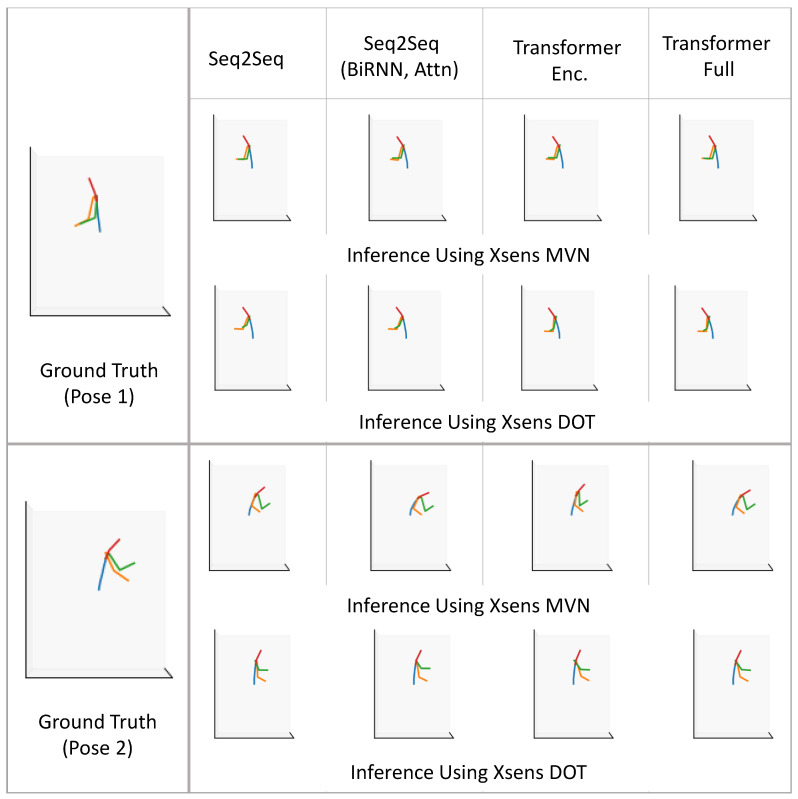
Qualitative evaluation for general human poses while standing (Pose 1) and sitting (Pose 2).

**Figure 25:**
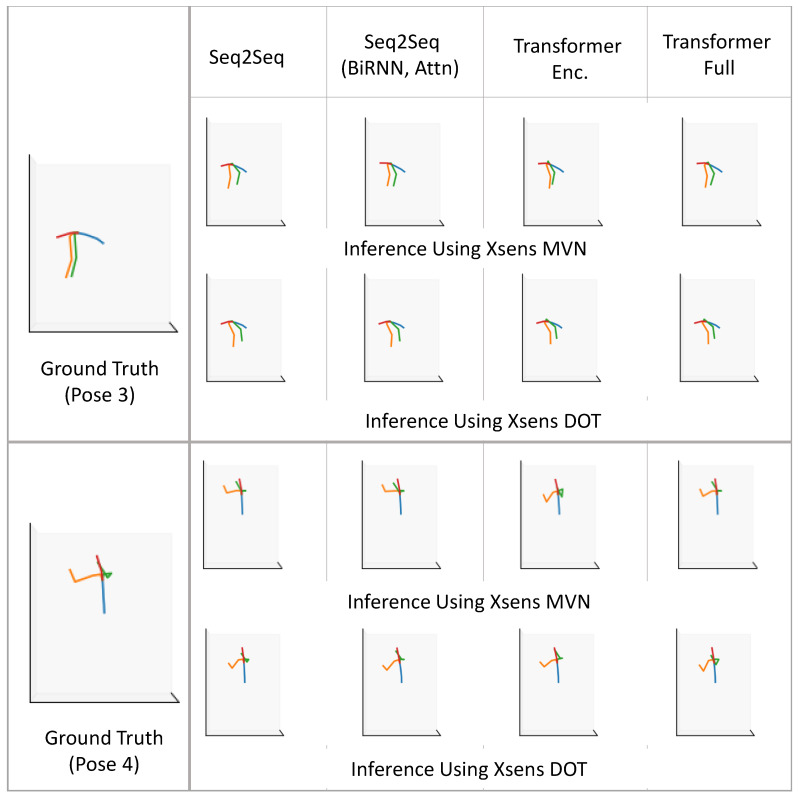
Qualitative evaluation is presented for challenging human poses while standing (Pose 4) and sitting (Pose 3). These poses are relatively challenging for inference since the person uses many hand or body movements.

In Figure 24 and Figure 25, we compare motion inference results using sparse segments of XSens MVN and XSens DOT (Configuration 1, variable mapping function). In each of the two figures, in the left-most column, we present the ground truth pose (as described in Figure 23), and on the right, we present inference results for both XSens MVN and XSens DOT from the four different machine learning models.

In the first pose (Figure 24 top), the person is standing and performing vacuum cleaning. Almost all the models performed well for both the XSens MVN and DOT sensors, giving reasonable-looking results. Therefore, we expect good inference results for similar tasks where the person will be standing and doing other activities of daily living such as washing dishes in the kitchen, making food, or cleaning. In the second pose (Figure 24 bottom), the person folds laundry while sitting in a chair. This pose is similar to sitting for a meal or working on a study table or similar environment where the person does not need to bend much. Both sensor types again gave reasonable results. In the case of DOT sensors, the left elbow was inferred to be slightly more open than in the ground truth. Both sensor types show the right upper arm to be rotated slightly relative to the ground truth.

The third and fourth poses (Figure 25) were more challenging than the first two poses. In the third pose, the person bends more than 90°. This is similar to tasks such as picking up objects from the floor or organizing low objects. All models performed similarly for both the XSens MVN and DOT sensors, and gave reasonable outputs. In all of the models, the arms are not as far forward as in the ground truth. In the fourth pose, the person was reaching upward. Pose 4 was similar to organizing objects on a shelf, grabbing grocery objects from a refrigerator, placing laundry items in the closet, or similar tasks. The transformer models did not perform as well for the DOT sensors, but overall all of the models performed reasonably. The MVN inference was slightly better than the DOT inference. Overall, the qualitative results resemble the quantitative evaluation of our models.

## 5. Discussion

### 5.1. Discussion on Localization

#### 5.1.1. Proximity Reporting

This work has developed model-free techniques for proximity reporting. The neural network takes as input RSS signatures from the beacons. During training, the neural network jointly learns the correlation among the beacons and the correlation between the target’s position and received RSS. This learning approach circumvents the need to derive RSS thresholds for each beacon. The learning approach is validated in terms of its ability to detect whether the simulated patient is within a predefined range from the anchor, its ability to detect when the simulated patient is not within a predefined range from the anchor, and its ability to either place the simulated patient within the range from the anchor or to determine the absence of the simulated patient within a certain range from the anchor. From the results presented in Figure 11a, all evaluated metrics deteriorate as the shadowing variance increases. This is intuitive, as the shadowing models the power fluctuation due to objects obstructing the propagation path between transmitter and receiver. A measurement from an obstructed beacon will most times have a reduced RSS value, giving the illusion that the target is much farther away than it actually is. This bias hampers any effect to accurately position the target. Figure 11 also depicts the advantage of accounting for the correlation between past and current measurements. The LSTM has better accuracy metrics than the DNN because it considers past measurements as well as future measurements when returning a proximity report.

#### 5.1.2. Positioning with Real World Data

Figure 13a and Figure 14a describe the time variation of the RSS from all of the anchors at the 1st reference point and 11th reference point, respectively. Clearly, the closest anchor has the highest mean RSS values. At the 1st reference point, the anchor with beacon ID 5 was the closest and had the highest mean RSS value (−69 dB). At the 11th reference point, the anchor with beacon ID 1 was the closest and had the highest mean RSS value (−63 dB). Figure 15a, Figure 16a and Figure 17a depict a few results showing the estimates and true positions. The estimates are roughly within the bounds of a room, which is likely sufficient for the interpretation of upper body kinematics. In [36], a multi-layer perceptron was used to achieve an accuracy of 2.82 m. In [97], a discriminant-adaptive neural network was developed for indoor positioning. A 23×30 m area was considered and a position accuracy of 2 m was achieved 60% of the time. In [28], a weighted *k*-NN approach was used for indoor positioning. For a similar 23×30 m area, an accuracy of 2 m was achieved 40% of the time. Considering all these works, our results also provide similar positioning accuracy. The positioning accuracy of our system varied from 1.3 m to 2.3 m.

### 5.2. Discussion on Motion Inference

Our results are well comparable to other previous work such as [57,59,61]. In [57], the authors used five sparse XSens MVN segments for predicting full-body poses, and compared six different configurations. Among them, configuration B was similar to our work. In configuration B, they placed sensors in the ‘pelvis’, ‘left forearm’, ‘right forearm’, ‘left lower leg’, and ‘right lower leg’ segments. The upper body in this configuration was comparable with our study (variable mapping with DOT sensor and sparse segment configuration of XSens MVN). Their estimates had an average joint angle error of ∼7° and joint position error of ∼8 cm for the full body, which is impressive. However, considering only the joints in the upper body, the mean joint angle errors were ∼12–15° (Figure 5 in reference [57]), using five sparse sensors. In [59], the authors predicted skinned multi-person linear model (SMPL, [98]) parameters of a single frame using 20 past frames and five future frames at test time with a bidirectional LSTM network. They performed both online and offline evaluations. From Table 3 in reference [59], for offline evaluation after fine-tuning, their model estimated mean (±standard deviation) joint angle errors of ∼16° ± 13° for the TotalCapture test dataset and ∼18° ± 12° for the DIP-IMU dataset. In the recent work in [61], authors also used the SMPL parameters, and they performed both localization and motion inference using six standalone IMU sensors. Looking at the results for offline comparison in Tables 2 and 3 in [61], they estimated a mean global rotational error of ∼12° ± 6° for the TotalCapture test dataset and ∼8° ± 5° for the DIP-IMU dataset. Although [59,61] list joint angle errors, these works use SMPL as a model, while we use segment orientation directly, which may lead to some differences in comparison. In all cases, there is a moderately large standard deviation.

All the works listed used five or more sensors to predict full-body motion, whereas our work uses three sensors to predict just the upper body. It may be that the upper and lower body halves function somewhat independently in their works, and would not affect their results if they just used the upper body and pelvis sensors. Our work found a mean segment orientation error of ∼15° using XSens MVN segments, and a mean of ∼20° using XSens DOT sensors for upper body inference. When we computed the joint angles (elbow and shoulder), we found mean average errors of <4° and standard deviations of 9–21°. These results are favorable as compared to previous works.

Furthermore, from Figure 21 and Figure 22, we find that joint angle distributions were similar to the ground truth. However, the segment orientations had a higher margin of error. This is because joint angles were computed as the angle between two segments. If the respective segments of inference equally deviate from the respective ground truth segments, the joint angle for inference and ground truth will be theoretically the same. Thus, looking at the segment orientation error will give more insight into a model’s performance.

Furthermore, we found that the forearms gave large errors with the DOT sensors but not the MVN sensors. This was confusing, since the inference was based on the sensors located on the forearms. It is likely that the forearm errors were caused by the DOT sensors drifting over time; the calibration mapping between the DOT sensors and machine learning model inputs were done once for each session, using a data frame near the beginning of the session. Thus, the DOT sensor drifting would result in errors since it would no longer match the true segment orientation. Surprisingly, the inference models seemed to be fairly immune to this drift in their estimation of the joint angles.

Overall, the DOT sensors did not perform as well as the MVN sensors. One reason for this is the imperfect mapping between the DOT sensors and the MVN system, which is what the machine learning models were trained on. The effects of the imperfect mapping are most evident when comparing the fixed mapping and the variable mapping. We found that the fixed mapping function did not perform very well at all (Figure 10 and Table 3). It turned out that the rotation matrices in Figure 10 for the individual calibrations varied substantially, with around 90° of rotation between two of them. It appears that, in general, a calibration must be performed for each individual, and again periodically over time as sensors move or drift. We note that the specific way the sensors will attach to a person’s forearm will likely differ somewhat between wearers, based on arm shape and variability in sensor placement, so a universal mapping may be difficult. With the MVN system, a full calibration was performed at the beginning of each data collection session, including special poses and walking for a short distance. The MVN system benefits both from this and also the presence of sensors on all body segments, which are used to solve the full skeleton.

As described before in Section 3.3.1, the XSens MVN uses 17 IMU sensors to reconstruct full body kinematics of 23 segments. MVN sensor reference frames are located inside the sensor (Figure 8a). However, the segment reference frames have a different location than the actual sensor locations. For example, segment frames for the left forearm and right forearm are located in the respective elbow joints. In comparison, we place the forearm sensors near the wrist (Figure 8e). Thus, the linear acceleration values were different for the segment and sensor. As the input to the model, we used segment orientation and experimented with both segment acceleration and sensor acceleration. We found that when doing MVN inference, using the segment acceleration gave better results (by 0.3–0.65°). However, when doing inference for the DOT sensors, using the sensor acceleration gave much better results (by 6–7° of mean segment angular error, and 2–7° of mean joint angle error). Since our ultimate goal was a standalone system with just a few IMUs, we ultimately used the sensor accelerations as inputs to our machine learning models.

It turned out that Configuration 1 (pelvis sensor on back of pelvis) performed better than Configuration 2 (pelvis sensor on the left hip), although the results were very comparable. As seen in Figure 20, Configuration 1 had mean errors about 1° less than Configuration 2 for all of the models. It may be that the sensor location in the back of the body moves with the pelvis more closely.

Overall, the kinematics estimated by this system do provide relatively large errors, as compared to whole-body IMU-based motion capture systems. However, this system is much easier to put on and is lower cost (<500 USD). It remains to be seen if the kinematic information is sufficient for rehabilitation applications; it is promising that the overall joint angle distributions were close to the ground truth distributions, and the average joint angle errors were small. Hopefully, with improvements, the overall trends in activity will provide insights into which upper extremity motions need additional rehabilitation.

### 5.3. Limitations of our Study

#### Motion Inference

Although we can predict upper-body motion with a reasonable error margin, there are some limitations and room for improvement in the future.

One easy way to improve the results with the DOT sensors is to increase their sampling rate. With the DOT sensors, the error increases with dynamic applications. We recorded data with the DOT sensors at a 60 Hz rate, but it would be better to record at a 120 Hz rate, which is recommended for dynamic applications.

Another limitation is that a custom calibration seems to be necessary for each person and possibly each data collection session. Since the fixed mapping with the DOT sensors did not work well, in the future, algorithms that automatically calibrate the sensor placement to a person are important to minimize the mapping error. We expect that these will need to be continuously updating algorithms that adjust even if a sensor moves over the course of a day, for example if a person takes off a wrist-mounted sensor and puts it back on again or if they disturb the orientation of the pelvis sensor. These algorithms should ideally also take into account any translational offsets between the MVN skeleton and wrist or pelvis, and thus improve the treatment of the acceleration.

## 6. Conclusions

In conclusion, we present several algorithms for in-home localization and kinematics reconstruction. We first present and simulate a new model-free technique for localizing a person to a region of interest. This is useful for identifying which room of a house a person is in. This technique employs a neural network to provide proximity reports based on the received RSS from beacons with known locations. Second, we validate the model-free proximity reporting by designing a neural network to localize a person to an RoI. Third, we conducted experiments validating a Bluetooth RSS fingerprinting-based approach to localization in a home environment. Finally, we presented algorithms for motion inference and data on how well three standalone IMU sensors can reconstruct the upper body kinematics. We compared two different configurations of the pelvis sensor, finding that they performed similarly. We also evaluated the possibility of a fixed mapping between the standalone IMUs and the MVN system used to train the machine learning models. We found that a calibration is necessary for each individual participant in order to get usable results. Once properly calibrated, the upper body inference system gave moderate segment orientation errors, but small mean errors for the joint angles.

It remains to be seen if the localization accuracy and the joint angle error accuracy are necessary for effective rehabilitation. It is likely important to have moderately-accurate human sensing so that the rehabilitation suggestions are based upon true data. While not explored in this paper, there may be other derived features (such as joint velocities) that may be especially useful for rehabilitation; it is unknown how well the algorithms presented here would accurately measure or estimate those. Overall, however, the work in this paper is promising for quantitative in-home rehabilitation.

## Figures and Tables

**Table 1:** Training parameters.

Parameter	Value
Learning Rate, α	0.01
Iterations (epochs)	400
Batch size, $T_{train}$	500
Training Shadowing variance, $\sigma_{s}$	7.5

**Table 2:** Important training parameters used in different deep learning models. In addition to these parameters, both the Transformer models used tuning parameters $\beta_{1}=0.95$ and $\beta_{2}=0.99$.

Parameters	Deep Learning Models			
	Seq2Seq	Seq2Seq (BiRNN, Attn.)	Transformer Encoder	Transformer Full
Batch Size	32	32	32	32
Sequence Length	30	30	30	30
Downsample	6	6	6	6
In-out-ratio	1	1	1	1
Stride	30	30	30	30
Hidden-Size	512	512	N/A	N/A
Number of Epochs	3	3	3	3
Dropout	0.1	0.1	0.1	0.1
Number of Heads	N/A	N/A	21	4
Number of Layers	N/A	N/A	2	4
Feedforward Size	N/A	N/A	200	2048

**Table 3:** Mean angular error of different models with varying configurations for inference using DOT sensors with the fixed mapping function (with the new test set).

Deep Learning Model	Fixed Mapping, Configuration 1	Fixed Mapping, Configuration 2
Seq2Seq	34.75°	44.85°
Seq2Seq (BiRNN, Attn)	33.07°	46.37°
Transformer Enc.	33.81°	42.92°
Transformer Full	32.65°	43.03°

**Figure A1:**
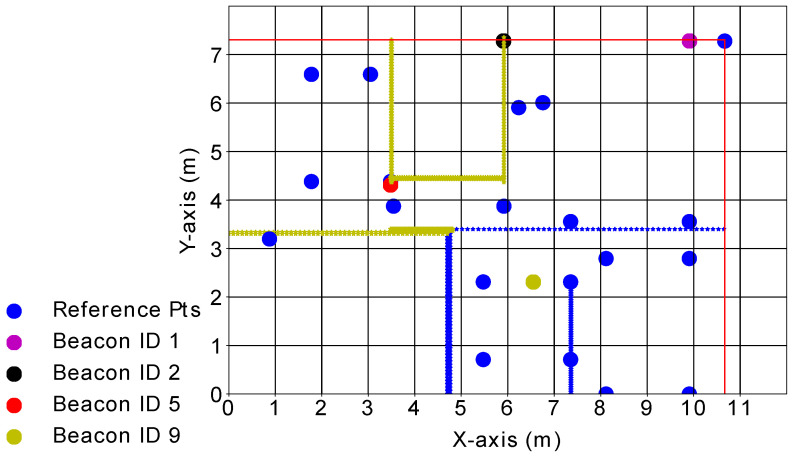
Positions of the anchors (beacons) used for transmitting Bluetooth signals, as well as the reference points where the signal strength was measured.

## Data Availability

Links to the Virginia Tech Natural Motion Dataset and code used to train our machine learning models can be found in [60].

## Appendix A. Detailed List of ADL Activities

Following is a detailed list of the activities performed during data collection for the kinematics reconstruction.

**Table A1:** List of activities performed by the new participants. The Activity Time is the approximate number of minutes to complete the activity.

Task Group	Room Preparation and List of Activities in Details	Activity Time
(1) Walking 3 Laps in the room	**Setup/Room Preparation:** Remove any objects from the ground. Make sure there are minimal obstacles in the room while walking. **Direction/Steps of activities:** Stay in N-pose for 10 s, then walk back and forth. First loop: Walk slowly from one room to another room and return to the starting location. Second loop: Walk in a medium speed from one room to another room and return to the starting location. Third loop: Walk in a faster speed from one room to another room and return to the starting location. Stay in N-pose for 10 s, then walk back and forth.	2.5
(2) Pick up things (clothing, books, toys, words blocks, safety pins) off floor, tidy up in order to vacuum, then vacuum.	**Setup/Room Preparation:** Place variety of items scattered across floor. Make sure vacuum is present and accessible. Initially 5 objects (book, toy, pen, cloth, coffee mug). **Direction/Steps of activities:** Pick 2/3 items from the floor, then place items on the coffee table. Pick remaining items from the floor, then place the items on the coffee table. Grab the vacuum cleaner, plug it in to the power source, and start vacuuming. Unplug the vacuum cleaner, then place it in its original location. Pick up all the five objects (one by one) from the coffee table and place them at their designated place. For example, the book will go to the bookshelf; the coffee mug will go to the kitchen shell, etc. Stay in N-pose for 10 s, then walk back and forth.	4
(3) Fold laundry and put it away in cabinets with appropriateabel/low drawers in multiple rooms (i.e., bedroom, linen closet, kitchen towels, hanging clothes, etc.)	**Setup/Room Preparation:** Prepare laundry basket. Include linens, hanging clothes, and folded clothes. These cloths will be placed in drawers, shelves, and linen closet labeled. **Direction/Steps of activities:** Take the laundry basket (full of 2 shirts, 1 T-shirt, 1 pillow cover, 1 pair socks) to the linen closet Sit in a chair. Then pick up the clothes and fold them. After folding each clothing item, place them in their designated location. For example, T-shirts will be hung on the hanger, linens will go to the linen closet, etc. Stay in N-pose for 10 s, then walk back and forth.	3.5
(4) Packing and unpacking a bag of groceries and put each piece in the cabinet/fridge with the appropriate label (by category).	**Setup/Room Preparation:** Need to place grocery items (5 items, e.g., a bag of coffee beans, jar of sugar, salt cellar, soda can, canned tuna) in the kitchen; also, a grocery bag should be accessible. Prepare/empty shelf space; label spots for the type of goods. **Direction/Steps of activities:** Take the grocery bag and load all items carefully into the grocery bag. Unpack the grocery items one by one and place them at their designated destinations. For example, the soda can will go to the fridge; the sugar jar will be placed in the kitchen cabinet. After organizing the groceries, fold the grocery bag and put it into a drawer. Stay in N-pose for 10 s, then walk back and forth.	2

## Appendix B. Anchor Characteristics

**Table A2:** Anchor characteristics for proximity reporting.

Beacon ID	Position (x,y)	Parameters $\left(\mathrm{PL}\left(d_{0}\right),\xi_{u},\sigma_{s,u}\right)$
1	(5,37.5)	$\left(-57.6,-2.5,\sigma_{s}\right)$
2	(15,37.5)	$\left(-68.3,-1.8,\sigma_{s}\right)$
3	(25,37.5)	$\left(-68.0,-1.9,\sigma_{s}\right)$
4	(35, 37.5)	$\left(-60.8,-2.3,\sigma_{s}\right)$
5	(45, 37.5)	$\left(-60.3,-2.4,\sigma_{s}\right)$
6	(5, 12.5)	$\left(-66.5,-1.9,\sigma_{s}\right)$
7	(15, 12.5)	$\left(-55.7,-2.6,\sigma_{s}\right)$
8	(25, 12.5)	$\left(-73.0,-1.2,\sigma_{s}\right)$
9	(35, 12.5)	$\left(-61.9,-2.0,\sigma_{s}\right)$
10	(45, 12.5)	$\left(-57.6,-2.6,\sigma_{s}\right)$
